# Organic functionality in responsive paramagnetic nanostructures

**DOI:** 10.3389/fchem.2025.1605538

**Published:** 2025-08-14

**Authors:** Anna M. Duncan, Connor M. Ellis, James P. Smith, Lillian Leutloff, Matthew J. Langton, Jason J. Davis

**Affiliations:** Department of Chemistry, University of Oxford, Oxford, United Kingdom

**Keywords:** MRI, nanoparticles, polymers, responsive, organic

## Abstract

Magnetic resonance imaging (MRI) has become an invaluable tool for diagnosing and monitoring a range of medical conditions, including cancer and cardiovascular disease, owing, in large part, to its high spatial resolution. Despite this, MRI suffers from an inherent low sensitivity, a drawback that can be mitigated through the use of exogenous contrast agents. Although molecular paramagnetic contrast agents are most commonly used, they suffer from significant limitations, including short circulation times, inadequate sensitivity, moderate (or no) tissue specificity, and potential toxicity. Recent advancements in nanomaterials research have paved the way for the development of paramagnetic nanoplatforms offering a promising alternative to these traditional chelates. Responsive contrast agents have gained attention due to their ability to generate local contrast in areas of particular interest, enabling the potential for disease-specific reporting where environmental factors including pH, ion concentration and biomolecule activity deviate from the norm. In addition to this, the generation of local or locality-specific contrast can help to overcome the intrinsic nonspecific nature of traditional contrast agents allowing for overall better treatment options. Purely organic nanoparticles, including those which are micellar, liposomal or dendritic and inorganic-polymer hybrids, can support step changes in MRI signal generation and its diagnostic potency by leveraging the specific and responsive characteristics of the organic components. This review seeks to illustrate how the integration of organic chemistry into magnetic nanostructures can enable responsive high-contrast generation.

## 1 Introduction

### 1.1 Magnetic resonance imaging

The detection of disease and early or associated physiological irregularities is crucial in improving health outcomes (Deulkar et al., 2024). It aids prophylaxis, enhances surveillance capabilities, and facilitates a more effective and personalised treatment (Cormode et al., 2009; Mura and Couvreur, 2012; Crommelin and Florence, 2013). The early detection of diseases such as cancer is, for example, highly correlated with the probability of recovery; for example, prostate cancer detected in stages 1, 2, or three presents with nearly a 100% 1 year survival rate, this falling to a 87.6% 1 year survival rate when detected in stage 4 (Hawkes, 2019). Although ∼50% of cancers are currently diagnosed at stages 3 and 4, a robust screening and improved imaging sensitivity can help physicians accurately determine the presence of a lesion, and if it is likely to be malignant (Crosby et al., 2022). MRI scans of high detail and edge resolution, in particular, yield valuable information on tumour morphology, and thus inform on the likelihood of malignancy. Methodologies that support the non-invasive visualisation of pathology include magnetic resonance imaging (MRI), positron emission topography (PET), single photon emission tomography (SPECT), computed tomography (CT), ultrasound (US), optical imaging (OI) and photoacoustic imaging (PAI) (Smith and Gambhir, 2017; Liu Y. et al., 2019; Walter et al., 2020; Hsu et al., 2023). The choice of imaging method needs to be tailored to a specific resource availability and clinical diagnostic aim; MRI is a powerful imaging modality with an anatomical spatial resolution of 1 mm and unlimited depth penetration but with an associated low sensitivity (µM–mM) (Alvares et al., 2017). To address this and add functionality, contrast agents (CAs) can be used to enhance diagnostic value through either increased generic signal:noise and/or environmentally-specific signal generation (see Section 1.2).

Based on nuclear magnetic resonance (NMR) principles, MRI analyses probe the interaction of magnetically active (non-integer spin quantum number) nuclei with an externally applied magnetic field (Jackson et al., 2021). Clinical MRI scanners facilitate the spatial mapping of water ^1^H (proton) signal density within various soft tissue structures of the body, depending on their specific magnetochemical environment, the latter having a dependence on the local concentration of water (Bley et al., 2010; Kostevšek, 2020). Crucially, this allows for the detection of structural abnormalities and disease, including, but not limited to sites of tumour (as noted above), injury and infection (Brindle, 2008; Abramovitch et al., 1998; Modic et al., 1986). Different classes of MRI imaging employ various scanner design, pulse sequences and image weighting. The majority of MRI scanners for diagnostic purposes are closed-bore systems, where the magnetic field is generated by passing an electrical current through a superconducting niobium-titanium (Nb-Ti) solenoid (<9.3 K) surrounded by copper (Busse et al., 2018; Warner, 2016; Zhang et al., 2019; Parizh et al., 2017). The generation of cross-sectional images necessitates the use of a strong (commonly 1.5–3 T) magnetic field to align the water proton spins (Berger, 2002); subsequently, longitudinal and transverse relaxation processes result in the restoration of equilibrium magnetisation as mapped by a Fourier transformation of the FID signals measured by the receiver (Edelstein et al., 1984). Subtle differences in the rates of these relaxation processes are reflective of local environment. The most commonly employed imaging sequences within MRI are *T*_1_-weighted and *T*_2_-weighted. The former utilise both short repetition times (TR) between successive pulse sequences and echo times (TE) between the delivery of the radiofrequency (RF) pulse and the reception of the echo signal, whilst the opposite is true for *T*_2_-weighted scans (Kawahara et al., 2021; Jung and Weigel, 2013).

**FIGURE 1:**
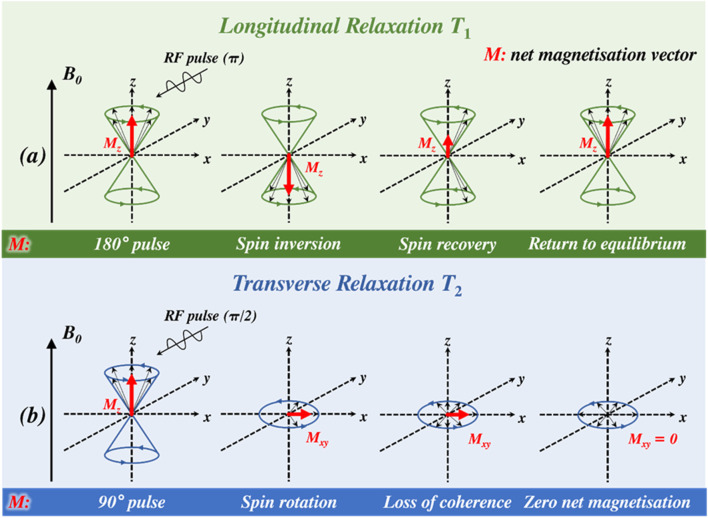
**(a)** Application of a 180^o^ (π) radiofrequency (RF) pulse inverts the net magnetisation vector (*M*_z_, illustrated by the red arrow) onto the -*z*-axis, with equilibrium restored by a spin-lattice relaxation process on removal of the RF pulse (time constant = *T*_1_). **(b)** A 90^o^ (π/2) RF pulse focuses spin, introducing a net magnetisation vector in the *xy*-plane (*M*_xy_) and a FID that decays to zero (with a time constant = *T*_2_) as spins lose coherence.

To elucidate the time constant of the longitudinal relaxation process (*T*_1_), a 180^o^ (π) RF pulse is used to re-orient the spins away from equilibrium, inverting their net magnetisation vector (*M*_z_) (Bain, 1990). Spin-lattice relaxation restores equilibrium magnetisation, with the rate of change along the z-axis (*M*_z_) described by a rate constant, *R*_1_, where *R*_1_ = 1/*T*_1_ (Williams et al., 2005; Spencer and Fishbein, 2000; Kingsley, 1999). To probe transverse relaxation, a 90^o^ (π/2) RF pulse is applied that focuses protic spin into the *xy*-plane, specifically introducing a net magnetisation vector (*M*_xy_) (Xu and Chan, 1999). Loss of spin coherence results in *M*_xy_ progressively falling to zero and the associated detected free induction decay (FID) signal exponentially decaying with a time constant *T*_2_ (Le Botlan and Ouguerram, 1997). These concepts are summarised in Figure 1.

### 1.2 Contrast agents

**FIGURE 2:**
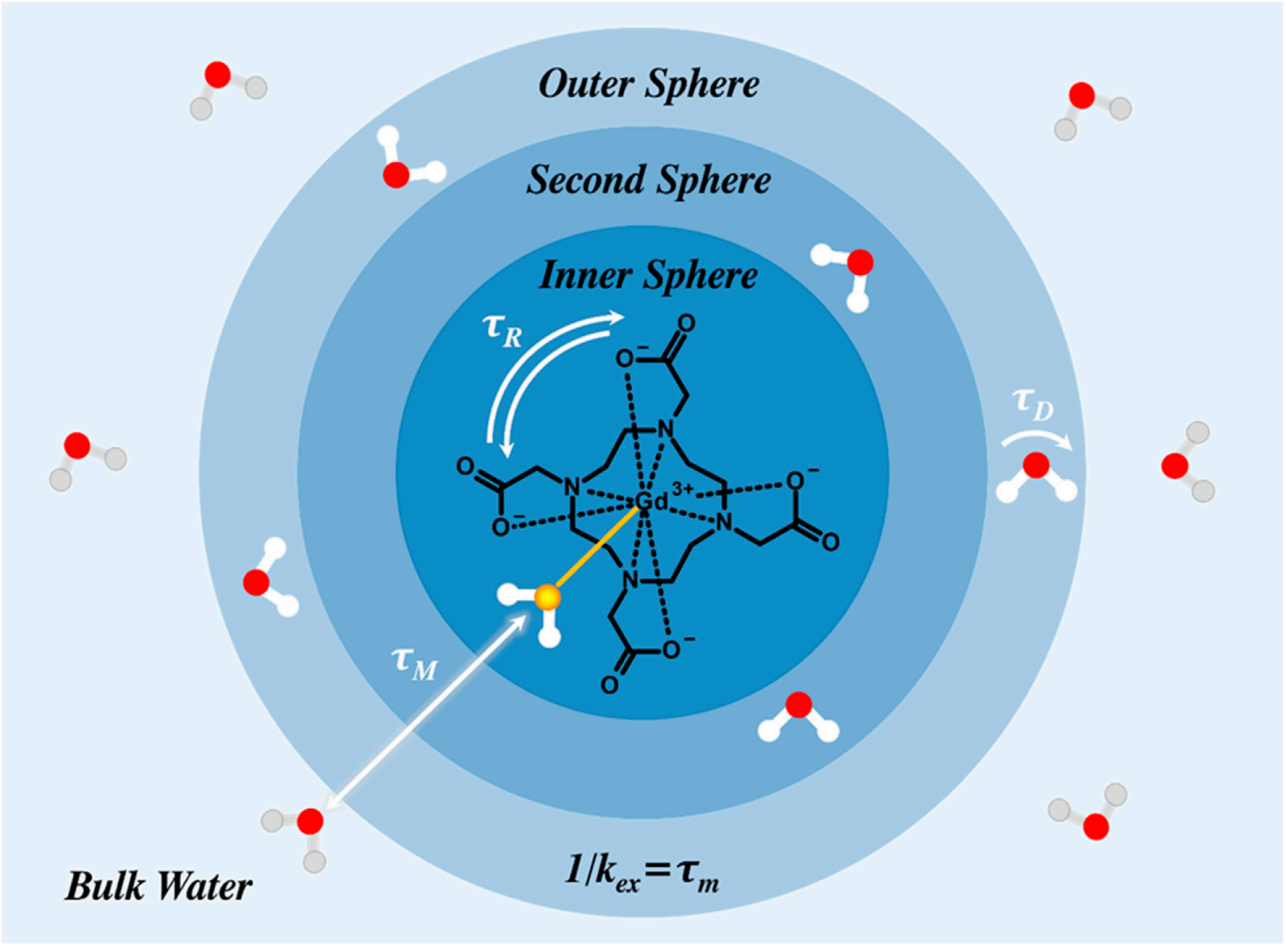
Water molecules can either coordinate directly with the paramagnetic centre (inner sphere), weakly interact with the chelating ligand (second sphere), or diffuse more freely (outer sphere). The rotational correlation time (or molecular tumbling rate) is denoted by *τ*_R_, the water exchange correlation time by *τ*_M_ (*k*_ex_ = water exchange rate), and the diffusion correlation time by $\tau_{D}$ .

MRI CAs allow an improved delineation (through differential image signal: noise) between different tissues, specific microenvironments, or anatomical structures within the body (De León-Rodríguez et al., 2015; Kim et al., 2000). The efficiency (effect per unit dose) of any given CA is defined by its relaxivity (Aime et al., 2009). Relaxivity, *r*_*i*_, measured in mM^-1^ s^-1^, is the linear gradient between the relaxation rate (*R*_*i*_ = 1/*T*_*i*_, where *i* = 1, 2) against CA concentration (mM) (Werner et al., 2008). A shorter *T*_1_ corresponds to a brighter image, therefore, if specific tissue possesses relaxation rates that are either too slow (*i.e.,* long *T*_1_), or too close to the relaxation times of water in neighbouring tissues CAs (commonly lanthanide-based, often containing chelated Gd^3+^) are introduced to improve the spatial resolution/clarity of the MR image (Li et al., 2019). A shorter *T*_2_ corresponds to a darkening of image contrast, with *T*_2_ CAs introducing local magnetic field inhomogeneities that lead to a decrease in signal intensity through promotion of the transversal relaxation pathway. Clinically, CAs that enhance longitudinal relaxivity are generally more useful than CAs that augment transversal relaxivity, as a darker scan area is also indicative of artefacts/clots (Na and Hyeon, 2009; Geraldes and Laurent, 2009). Notably, a high relaxivity also means that a lower dose of the CA can be administered, reducing off-target effects (Lancelot et al., 2020; Jacques et al., 2010). The rational design of CAs with improved relaxivity generally considers a programmed slowing of molecular tumbling (by virtue of the increased size of the probe) and/or enhanced inner/second/outer sphere effects through either increased hydration, conformational rigidification, or an increased viscosity of surrounding water molecules (Figure 2). (Botta and Tei, 2012)

$\text{As noted},T_{1}$ CAs typically contain paramagnetic ions such as Gd^3+^ (gadolinium-based CAs, GBCAs) and are commonly used to enhance the visualization of blood vessels, tumours, inflammation, and specific organs (Blomqvist et al., 2022; Iyad et al., 2023). They consist of kinetically stable Gd-chelates and include the use of ligands such as 2,2′,2″,2‴-(1,4,7,10-tetraazacyclododecane-1,4,7,10-tetrayl)tetraacetic acid (DOTA) and *N*,*N*′-[(Carboxymethyl)azanediyl]di (ethane-2,1-diyl)bis [*N*-(carboxymethyl)glycine] (DTPA) (Bousquet et al., 1988). Low molecular weight high spin complexes of Fe^3+^ and Mn^2+^ can also be used as lower relaxivity substitutes but are, additionally, accompanied by not insignificant toxicity concerns (Wang et al., 2019; Manavalan et al., 2024; Devreux et al., 2021; Drahoš et al., 2012).

Many such Gd-based agents carry the risk of nephrogenic systemic fibrosis (NSF) resulting from the de-chelation of Gd^3+^ from its associated macrocycle, primarily affecting patients with compromised renal function (Ersoy and Rybicki, 2007; Lin and Brown, 2007). Gadoterate (Gd-DOTA) and gadoteridol (Gd-HP-DO3A) have been found to be the most stable CAs, possessing minimal susceptibility to de-chelation, with thermodynamic stability constant (log K_therm_) values of 25.6 and 23.8 respectively and associated favourable half-lives (*T*_1/2_) of 338 h and 3.9 h (Hao et al., 2012). One safety precaution that is taken to protect against de-chelation during transport and storage, and minimise acute toxicity, involves adding excess chelate (0.23 mg/mL in the case of Gd-HP-DO3A) to minimise transmetallation following intravenous (IV) administration (Morcos, 2008). A reduction in serum calcium upon IV injection has been observed for less kinetically stable Gd-chelates, such as gadodiamide (Gd-DTPA-BMA) and gadoversetamide (Gd-DTPA-BMEA); this is known as ‘spurious hypocalcaemia’ and occurs with a concomitant (long-term) deposition of Gd^3+^ in the bone (Kirchin and Runge, 2003). This is not, however, observed for Gd-DOTA and Gd-BT-DO3A (gadobutrol), which are regarded as well-tolerated (Normann et al., 1995). It is worth noting that a connection between Gd^3+^ bone deposition and adverse side effects has so far not been confirmed (Lapusan et al., 2024), but, nonetheless, careful consideration should be made when reviewing the use of Gd-based CAs in pregnant and paediatric patients especially (Alghamdi, 2023).

$T_{2}$ CAs typically employ superparamagnetic materials, such as iron oxide nanoparticles (IONPs) which induce local magnetic field inhomogeneities in the vicinity of the probe, resulting in decreased signal intensity within *T*_2_-weighted images (Jeon et al., 2021). IONPs are generally coated with a biocompatible and colloid-stabilising functionality, such as that presented by a passivating polymer coat that includes poly (ethylene glycol) (PEG) (Rezaei et al., 2024; Lazaro-Carrillo et al., 2020). Indeed, IONPs are routinely surface modified with PEG to impart improved blood circulation times (See Section 2). (Zhang et al., 2024) Such particles are commonly used in visualisation of liver tissue and in the detection of lymph node metastases because these tissues efficiently uptake the particles by macrophages (Yan et al., 2023; Gaharw et al., 2019). They are generally well-tolerated (minimal to zero side effects), but rare serious adverse reactions such as anaphylaxis have been reported (Pellico et al., 2023).

It should be noted that many $T_{1}$ and/or $T_{2}$ MRI active scaffolds been explored to improve contrast probe relaxivity and biocompatibility, including wholly inorganic particles (such as mesoporous silica nanoparticles (Taylor et al., 2008), metal organic frameworks (Bunzen and Jirák, 2022), and gold nanoparticles (Zeng et al., 2014)), purely organic nanomaterials (such as lipid vesicles (Langereis et al., 2013) and polymer micelles (Nasongkla et al., 2006)), and hybrid nanoparticles thereof (Arosio et al., 2013). The incorporation of paramagnetic chelates within a nanoparticle platform is an attractive way of modulating several Solomon-Bloembergen-Morgan (SBM)-governing parameters, such as those introduced in Figure 2, that are responsible for large baseline boosts in relaxivity (Wahsner et al., 2019). The anchoring of a Gd-chelate to a rigid or semi-rigid scaffold such as a inorganic nanoparticle or a polymer chain restricts the rotation of the chelate, stretching $\tau_{R}$ , and typically moving the characteristic rotational frequency ( $1/\tau_{R}$ ) closer to alignment with the Larmor frequency of water (notably improving relaxivity) (Verwilst et al., 2015; Pellico et al., 2019a). Similarly, the confinement of a paramagnetic chelate within a nanoparticle scaffold, *e.g.*, entrapping within a polymer matrix or porous inorganic nanoparticle, can lead to refined SBM parameters, such as restricted water exchange rate ( $\tau_{M}$ ), increased rotational correlation time ( $\tau_{R}$ ), and enhanced local water hydration (*i.e.*, amplified outer sphere effects) with the central lanthanide, effects also able to support an improved relaxivity (Villaraza et al., 2010). These modified nanoparticulate scaffolds are generally metabolised in the liver, the main detoxification organ, with associated potential biosafety concerns (Wang et al., 2024). Inadequate elimination may result in the long-term accumulation of such nanomaterials in hepatocytes, impairing their normal biological function. Harmful downstream effects may also result from the production of reactive oxygen species, associated destructive interactions with DNA/mitochondria, and effects on intracellular signalling pathways; a range of *in vivo* and *in vitro* hepatotoxicity studies have now taken place in an attempt to understand and mitigate these effects (Yao et al., 2019).

### 1.3 Responsive organic contrast

The pathophysiology accompanying many diseases can result in altered chemical environments in affected and surrounding tissues; malignant tumours possess, for example, acidic extracellular pH levels (by virtue of a high rate of aerobic glycolysis, increasing lactic acid production) and hypoxia (through overproduction of, *e.g.,* glutathione (GSH) and cysteine) (Chen et al., 2020; Hao et al., 2018), with decreased tissue pH also found in conditions such as atherosclerosis (Naghavi et al., 2002) and renal disease (Kraut and Kurtz, 2005). Low extracellular pH is, thus, a common motivator in the rational design of bio-responsive particles (Kim et al., 2000; Wu and Zhao, 2013). The use of a stimulus-responsive imaging probe is, in general terms, a significant diagnostic asset in supporting disease-specific reporting (Neves and Brindle, 2006). The integration of organic chemistry enables a tailored rational design (with characteristics engineered to respond to a physiological condition of interest), and improved relaxometric features within a facile chemical tunability (Davies et al., 2013; He et al., 2019; Matsumoto and Jasanoff, 2008; Priya James et al., 2014; Vithanarachchi and Allen, 2012). The so-generated environmentally-responsive CAs can enable localized contrast enhancement, activated at either a desired site or condition, and offer a spatial and temporal modulation of contrast (*e.g.*, dynamic imaging as the responsive CA modulates upon exposure to an altered tissue environment) (Foster and Larsen, 2023). External stimuli such as those associated with pH, biomolecule presence, light, ion presence, specific temperature, or redox agent activity can be leveraged to interact with this CA functionality such that contrast is switched ‘on’ in the presence of a particular environmental condition *in vivo* (Ge and Liu, 2013; Zhang Z. et al., 2022). Early responsive, non-particulate, MRI contrast focused on the design of simple macrocycles that contained responsive side arm modifications which influenced q (hydration number) through differences in chelation state on exogenous ion addition (Viswanathan et al., 2010). Specifically, side arm ion-association can result in a conformational change that introduces a vacant coordination site at the paramagnetic centre. In recent work, responsive organic functionality has been leveraged to modulate more subtle SBM-governing parameters, such as the rotational correlation time ( $\tau_{R}$ ) through locally triggered changes in the size of the nanoparticle agent, *e.g.*, by polymer swelling/deswelling or through altered colloidal properties with deviations in local pH (Cazares-Cortes et al., 2017). Similarly, water exchange rates ( $\tau_{M}$ ) can be manipulated by triggering changes in polymer hydration or chelate accessibility to bulk water within an organic agent (De Sarno et al., 2019).

**FIGURE 3:**
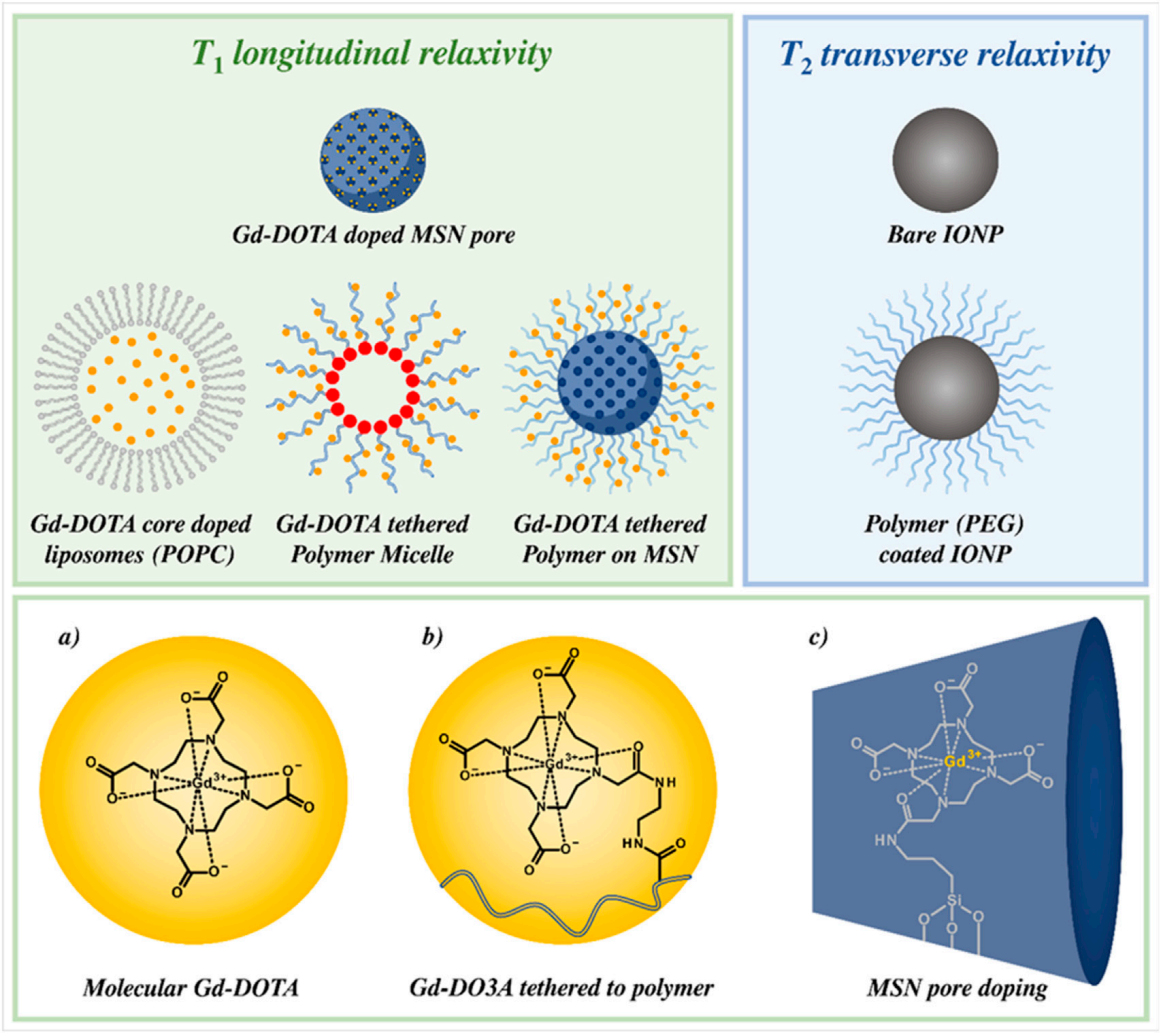
Overview of organically-tuneable IONPs, MSNs, artificial phospholipid vesicles (liposomes) and polymer micelles. *T*_1_ relaxivity (examples in green) is achieved through doping/tethering MR-active paramagnetic chelates that include Gd-complexes with a nanoparticle architecture. Displayed are: **(a)** molecular Gd-DOTA, **(b)** Gd-DO3A tethered to a polymeric strand, and **(c)** a doped MSN inner pore. The beneficial elongated rotational correlation characteristics associated with this integration enhance associated image contrast. Iron oxide-based particle platforms are used to promote *T*_2_ relaxation (blue), and can be wrapped in polymers (*e.g.,* PEG) to improve probe biocompatibility and/or elongate blood circulation times.

Polymers are commonly employed in the design of responsive MRI CAs, either as wholly polymeric nanoparticles that include polymer micelles, or as coatings for inorganic/organic nanostructures such as mesoporous silica nanoparticles (MSNs) and IONPs (Elsabahy et al., 2015; Gao et al., 2011). The former micelles are organic nanoparticles composed of amphiphilic polymer strands that undergo self-assembly above a critical micellar concentration (CMC) (Zhang and Zhao, 2016), whilst polymer coatings (such as PEG/PAA block copolymers) can be attached to nanoparticles such as MSNs, either through covalent functionalisation or electrostatic association (Pellico et al., 2019b). Responsive agents can also be generated from organic liposomes, phospholipid-based spherical NPs with a hollow core (and an ability to internally encapsulate MRI active cargo and/or drug); these have been explored as an (organic) responsive alternative to traditional MRI CAs, owing to their high chemical tuneability, versatility and good biocompatibility (Nsairat et al., 2022; Hingorani et al., 2015). A range of such polymer-inorganic hybrids and organic-based particles are summarised in Figure 3, with their operational mechanisms detailed in Sections 2,3.

The organic coating of MRI-active nanomaterials can have a profound effect on circulation time and practically-realistic contrast generation. The aggregation of NP CAs *in vivo* predictably correlates inversely with circulation lifetimes, strongly affects relaxivity, and is therefore a core consideration in design (Ta et al., 2017; Ahl et al., 1997). In the case of IONPs, aggregation takes place when interparticle distance is decreased and dipolar interactions increased, a process that may be facilitated by the desorption of capping ligands such as oleic acid (Roca et al., 2009). These aggregates, although possessing enhanced *r*_2,_ are more susceptible to uptake by macrophages. Similarly, aggregation of Ln-doped MSNs, for example, has an observed detrimental effect on their intrinsic relaxivity, due to the blocking/obstruction of the CA-containing pore (or surface) bound sites (Rizzi et al., 2021). In the case of MSNs, aggregation can also occur by protein-mediated neutralisation in serum, a process that is known to be circumvented by surface functionalisation with PEG or zwitterionic polymers (Liu et al., 2015; Lowe et al., 2015). In comparison, aggregation of liposomal structures (see Section 1.3 for discussion of stimuli-responsive liposomal CAs) can be induced by Ca^2+^ and Mg^2+^ (Rahnfeld et al., 2018). Cholesterol or phosphatidylglycerol containing liposomes are more rapidly cleared by the reticuloendothelial system, although glycolipids or polymers can be incorporated with the phospholipid structure to mitigate this (Ahl et al., 1997). Suitable organic coatings, then, reduce the prevalence of aggregation and support favourable circulation times *in vivo*, and have accordingly play a critical role in MRI CA design, regardless of any additional responsive functionality.

## 2 Paramagnetic polymer-inorganic hybrid nanoparticles

### 2.1 Polymer functionalised iron oxide nanoparticles

**FIGURE 4:**
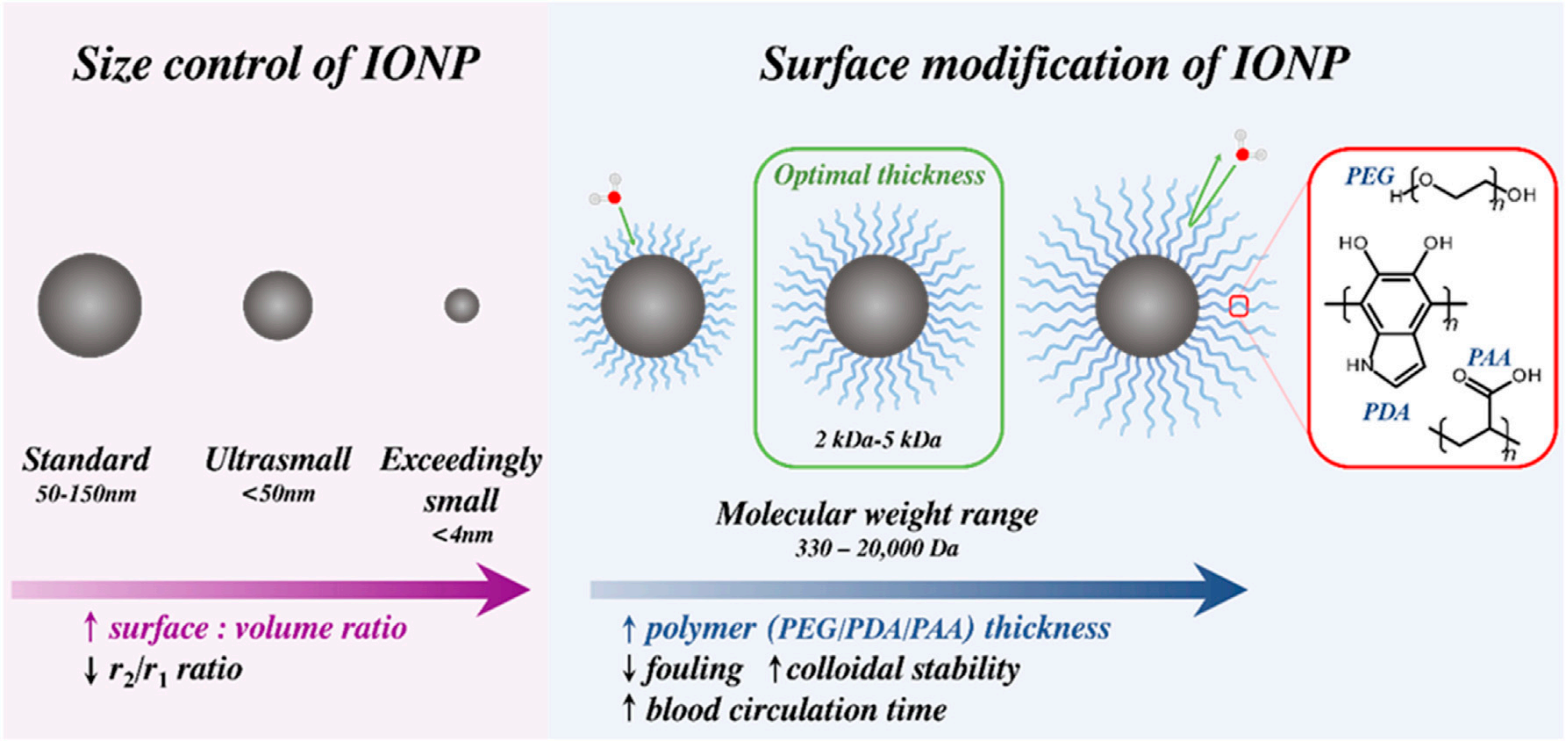
Overview of size effects and IONP surface modification. As diameter decreases, the increased surface effects mean that exceedingly small IONPs are suitable *T*_1_ CAs. Modification with hydrophilic polymers (*e.g.*, polyethylene glycol, PEG; polydopamine, PDA; polyacrylic acid, PAA) improves biocompatibility, blood retention time and colloidal stability, assisting clinical translatability.

Of the eight iron oxides known, magnetite (Fe_3_O_4_) and its oxidised form maghemite (γ-Fe_2_O_3_) are, in nanoparticulate form, common imaging probes which are routinely surface modified with biocompatible polymers such as PEG, sodium alginate, and poly (acrylic acid) (PAA) to improve colloidal stability under aqueous conditions (Dulińska-Litewka et al., 2019; Estelrich et al., 2015). There are many reported applications of IONPs, including the targeted delivery of therapeutics and for disease treatment through a considered exploitation of their magnetic properties, *e.g.,* in magnetic hyperthermia which promotes cell apoptosis in tumour tissue (Dadfar et al., 2019; Vallabani and Singh, 2018). Their use as imaging probes has been heavily analysed over the past few decades, and continues to receive interest due to their high magnetic moments, allowing for effective contrast generation at comparatively small dose. The saturation magnetisation of these is also known to be a function of morphology (size, structure and shape), and octahedral, tetrahedral, cuboid, plate and wire-like (*etc*.) IONPs have been successfully produced, building upon the traditional spherical structures (Xie et al., 2018). These geometric variants also have specific biocompatibility and bioclearance characteristics. Renal clearance, in general terms, has been shown to be ineffective for IONPs with diameters >50 nm, resulting in accumulation *in vivo* over extended timeframes, a clear problem if repeat MRI investigations are required (Lapusan et al., 2024). Superparamagnetic iron oxide based nanoparticles have also been employed to enable $T_{1}$ contrast generation, an ability ascribed to increased surface Fe^3+^ exposure, supressed magnetisation values and surface effects that influence both magnetisation and water exchange (see Figure 4). (Wei et al., 2017; Bao et al., 2018) The reduced size of IONPs is associated with significantly improved blood half-lives (e.g., 10–14 h for ferumoxytol, used for vascular imaging) due, in large part, to their reduced rate of opsonisation compared to larger diameter equivalents (e.g., 8 min for ferumoxide) (Lapusan et al., 2024).

Despite their ease of fabrication and favourable magnetic characteristics, the fouling of IONPs by protein within the blood/extracellular fluid onto the NP surface typically results in facile aggregation of these particles, leading to their quick removal by macrophages in a process known as opsonisation (Suk et al., 2016). To mitigate this, IONPs are routinely surface functionalised at the point of synthesis by hydrophilic entities such as PEG/PDA/PAA (as shown in Figure 4), a process which also improves their shelf-life for *in vivo* MRI applications, again aiding their clinical translatability (Lapusan et al., 2024).

PEG modification is a particularly common means to improve blood circulation time, colloidal stability, and biocompatibility (Feng et al., 2018). These effects are commonly referred to as ‘stealth’ and reduce the rapid clearance of native particles by the reticuloendothelial system. There has been much work investigating the optimal thickness of the stabilising PEG coat on the surfaces of different sized iron oxide NPs, with molecular weights typically ranging from 2 kDa to 5 kDa. For example, Larsen *et al.* systematically varied the molecular weight of the PEG-coating of IONPs, ranging from 330–20,000 Da, to investigate the effect on blood circulation time and macrophage uptake (Larsen et al., 2012). In this work, the larger molecular weight (20 kDa) PEG-IONPs were observed to possess the longest blood circulation times (45 min) whereas the low molecular weight (330 Da) PEG-IONPs exhibited the fastest macrophage-based removal. PEG-IONPs with a 5 kDa polymer coat displayed the best contrast generation with an associated $r_{2}$ = 354 mM^-1^ s^-1^ at 3 T, in data that indicated that thick polymer coatings can detrimentally impact associated relaxivities by restricting the approach of water (and therefore its effective dephasing). In similar work, Leal *et al.* reported the synthesis of PEG-IONPs with a variety of molecular weights ranging from 600 Da to 8 kDa, with the optimal PEG thickness reported to be 3,000 Da (associated $r_{2}$ = 151.1 ± 1.7 mM^-1^ s^-1^ at 9.4 T), conclusions mirrored in observations by Leal et al. (2015), Ramniceanu et al. (2016). These coating-optimised characteristics have also been observed with other polymers as illustrated, for example, by Cheah *et al.* where polydopamine (PDA), PAA, and PEG coated IONPs of tuneable sizes were investigated (PDA $M_{n}$ = 189.6 Da, PEG $M_{n}$ = 5,000 Da, and PAA $M_{n}$ = 1800 Da) (Cheah et al., 2021). All polymer-modified IONP formulations in this work exhibited good colloidal stabilities as resolved by dynamic light scattering (DLS), with the PAA-modified particles in particular displaying effective $T_{2}$ contrast enhancement ( $r_{2}$ = 75.3 mM^-1^ s^-1^ at 0.5 T).

While PEG and comparable polymers do impart a ‘stealth’ capacity for IONPs, a review of more than 5,000 publications on these particle systems by Wen *et al.* revealed that some 85% exhibited a rapid drop to half the original blood concentration within an hour of administration (Wen et al., 2023). This finding suggests that more investigation needs to be done into PEG alternatives, such as modified lipids, or co-polymeric polymersomes (Carol et al., 2015; Qi et al., 2018). Such advancements would allow for the concentration of the CA to remain high through clinically-relevant periods, allowing for a lower initial dose. Current research aligns with this trend, with an increasing number of organic polymers now applied in the post-synthetic surface modifications of IONPs. Alternative organic functionalities, such as those provided by dendrimers or polymer micelles, are also now commonly employed as IONP protective coatings (Salimi et al., 2018). While these studies show promise in addressing the systemic challenge of rapid clearance, further refinement is necessary to develop scalable, high contrast, and clinically approved agents with a true ‘stealth effect’ (Salimi et al., 2018).

**FIGURE 5:**
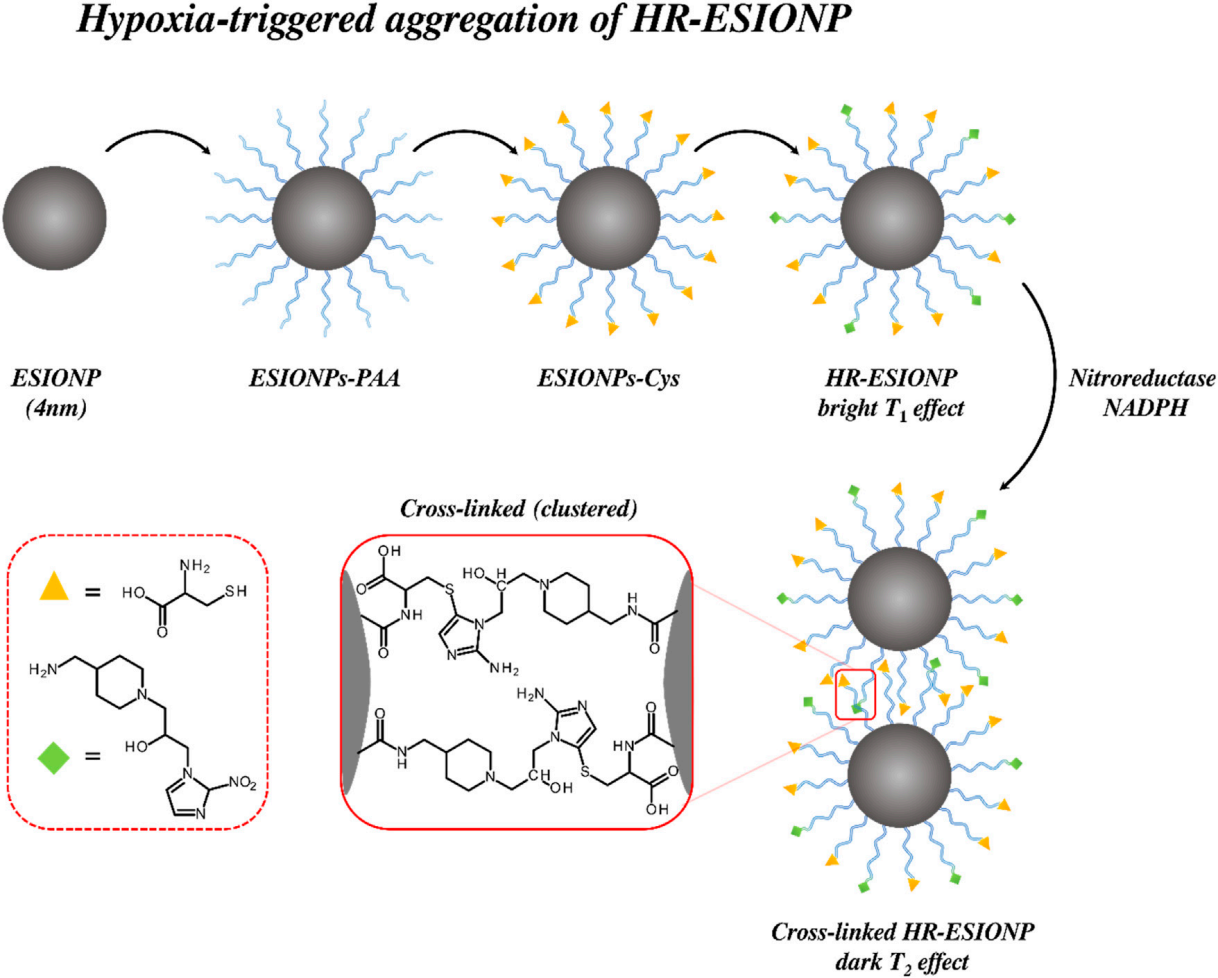
*T*_2_ contrast enhancement for polymer-modified “extremely small” iron oxide nanoparticles (ESIONPs) through cross-linked hypoxia triggered aggregation. In this work ESIONPs are coated with PAA and functionalised with both cysteine and nitroimidazole derivatives that result in an enhanced *T*_1_ signature. Upon exposure to nitroreductase and NADPH, cross-linking (clustering) occurs, resulting in an increase in *r*_2_ and *r*_2_/*r*_1_.

To achieve environmental-responsivity, an engineered triggered IONP aggregation can be employed to engender significant switches in $r_{2}$ . Lu *et al.* for example*,* have designed extremely small iron oxide nanoparticles (ESIONPs) with a diameter less than 4 nm, particles that typically exhibit *T*_1_ contrast in their monodispersed native state (Lu et al., 2023). These particles were modified with polyacrylic acid (PAA) to improve dispersity in aqueous environments, and subsequently functionalised with nitroimidazole and cysteine derivatives, moieties capable of cross-linking in the presence of nitroreductase and NADPH, frequently present and overexpressed in hypoxic tumours as shown in Figure 5 (Yang et al., 2017). The PAA coating on the ESIONPs enabled prolonged circulation and tumour site accumulation prior to this triggered aggregation.

Relaxivity measurements were taken at 0.5 T and 35 °C in PBS to examine the properties of the particles before and after exposure to a reducing environment. Prior to aggregation, the particles exhibited an *r*_2_ relaxivity of 31.81 mM^–1^ s^–1^, and an *r*_2_ */r*_1_ ratio of 1.66 (ratiometric analyses facilitating concentration independent assessments) (Hagberg et al., 2013). Upon treatment with NADPH and nitroreductase in a hypoxic environment, *r*_2_ was observed to increase to 141.21 mM^–1^ s^–1^, and the *r*_2_ */r*_1_ ratio to 7.81 ( $∆r_{2}$ = 109.4 mM^-1^ s^-1^). The shift in contrast-generating properties reflect the role of aggregation and clustering in dampening *r*_1_ relaxivity and increasing *r*_2_. To examine *in vivo* performance, the particles were injected into mice bearing a 4T1 xenograft tumour, mimicking human breast cancer progression. The image intensity supported by the responsive particles was compared to that offered by the non-responsive particles, with the former darkening (and accumulating) in hypoxic tumour areas. The combination of *T*_1_-to-*T*_2_ contrast switching, and dark contrast enhancement meant these agents, then, acted as effective hypoxia-sensitive CAs. These studies, in combination, have exemplified the use of suitable organic functionality, introduced onto the surface of IONPs through both grafting ‘to’ and ‘from’ approaches, in modulating IONP aggregation in a manner that is both tuneable and clinically pertinent.

### 2.2 Polymer-supported MSNs

MSNs have gained significant popularity as a platform for the delivery of a variety of payloads, including drugs and contrast agents (Mohamed Isa et al., 2021), possessing several attractive physicochemical features including good colloidal stabilities and facile chemical surface tuneability (Wu et al., 2011). They can be readily functionalised with chemical moieties through post-synthetic silanol surface modification, including that where polymers are introduced to yield a passive solubilising coat, active targeting capability, or groups susceptible to protonation/deprotonation that support stimuli-responsivity (Yuan et al., 2020). In comparison to other nanoparticulate formulations, MSNs show particular clinical utility due to their good colloidal stability in the bloodstream, low toxicity and facile interfacial functionalisation; and characteristics that support complementary delivery and tumour-targeting capabilities (Lérida-Viso et al., 2023). Silica based nanoparticles already have an established history in cancer imaging, with proven efficient clearance via hepatic and renal pathways, reinforcing their clinical translatability as a versatile platform (Benezra et al., 2011). From an MRI perspective, however, further studies are needed to comprehensively evaluate *in vivo* Gd^3+^ leaching kinetics from pore-doped MSNs (Carniato et al., 2018).

MSNs are typically coated with polymers in two distinct fashions, either through direct covalent binding or by electrostatic association. Covalent approaches most typically involve amide coupling or a surface-initiated polymerisation, with electrostatic assembly invoking supramolecular interactions between the charged portion of the polymer and the silica surface. Sodium alginate is, by way of example, a natural hydrophilic polysaccharide that has been widely employed for bio-responsive applications due to its negligible toxicity and high biodegradability (Sarkis et al., 2017). Li *et al.* have, for example, employed the aforementioned electrostatic association approach to yield dye cargo-entrapped gadolinium-doped MSNs (generated by an *in situ* doping of the MSN surface with Gd^3+^) coated with sodium alginate (Li Z. et al., 2022). Loss of electrostatic association between the MSNs and the alginate cap at a pH below the ${pK}_{a}$ of the alginate carboxylate groups ( ${pK}_{a}$ < 4.5) resulted in both triggered release of the fluorescent rhodamine B (RhB) dye and restored water access to the surface-doped paramagnetic Gd^3+^ ions. The latter displayed a corresponding five-fold switch in relaxivity, with $\Delta r_{1}$ = 40.57 mM^-1^ s^-1^ (at 0.5 T), of clinical note given that low pH is associated with endosome and lysosomal environments.

A very similar irreversible capping approach was adopted by He *et al.*, generating pH-responsive poly (*N,N-*dimethylacrylamide-co-THPA-functionalised *N-*(3-aminopropyl)methacrylamide) and poly (allylamine hydrochloride) surface coated MSNs loaded with Gd_2_O_3_ nanoparticles (He et al., 2019). As the pH decreases below pH 5.0, the poly (allylamine hydrochloride) protonates, reversing the previously favourable supramolecular association of the polymer to the charged particle surface and exposing the MSN pores. In doing so, the MR-active integrated Gd_2_O_3_ particles were, again, released, resulting in a corresponding switch in $r_{1}$ with $\Delta r_{1}$ = 5.71 mM^-1^ s^-1^ (at 3 T) as pH decreases from pH = 7.4 to pH = 5.0.

**FIGURE 6:**
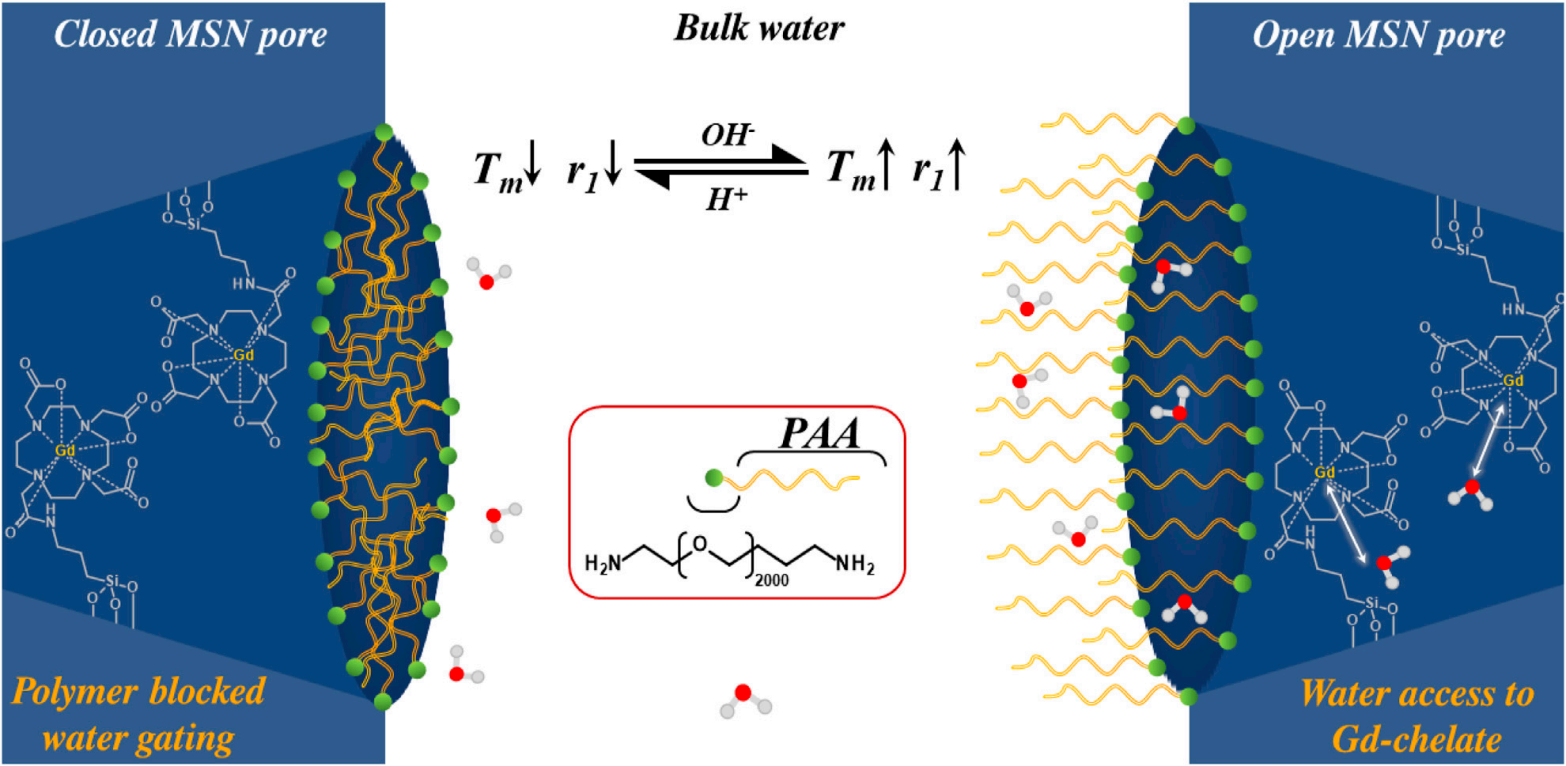
A schematic illustrating how water accessibility to integrated Gd-chelates within MSN mesopores can be reversibly gated with pH-responsive polymeric strands (specifically in this example by PAA). Deprotonation of the carboxylic acid groups within the polymer results in a conformational switch from globular to extended as individual negatively charged strands repel, improving water exchange within the pore channels (white arrows) and boosting $r_{1}$ by > 130%.

The above examples are indicative of potentially large ( $\Delta r_{1}$ > 150%) triggered responses but are, in essence, irreversible. With covalently modified particles, the permanence of the polymer coating can support partially or fully reversible relaxivity modulations. When designing a particle that is covalently modified with a polymer, changes to the conformation and chemical character of the organic layer can be a potent means to generate *reversible* relaxivity switches. One approach was demonstrated in work by Pellico *et al.* who synthesised MSNs covalently functionalised with PEG/PAA block copolymers (Pellico et al., 2019b). The use of a block copolymer enabled improved colloidal stability through the PEG block while also eliciting a pH-responsive, in this case a fully reversible, switch in $T_{1}$ imaging capabilities through the PAA block. This assumes a globular conformation below pH 4.8, resulting in a tight capping of the MSN pores and reducing diffusive water access to integrated Gd-motifs, diminishing relaxivity. As illustrated in Figure 6, above pH 4.8, individual polymer strands extend into solution (Sarkar and Somasundaran, 2004), significantly improving water accessibility to the integrated paramagnetic complexes and inducing a significant, and reversible, >130% switch in *r*_1_ (Δ*r*_1_ = 11.4 mM^-1^ s^-1^) as pH is traversed from pH 3.0 to pH 10.0.

**FIGURE 7:**
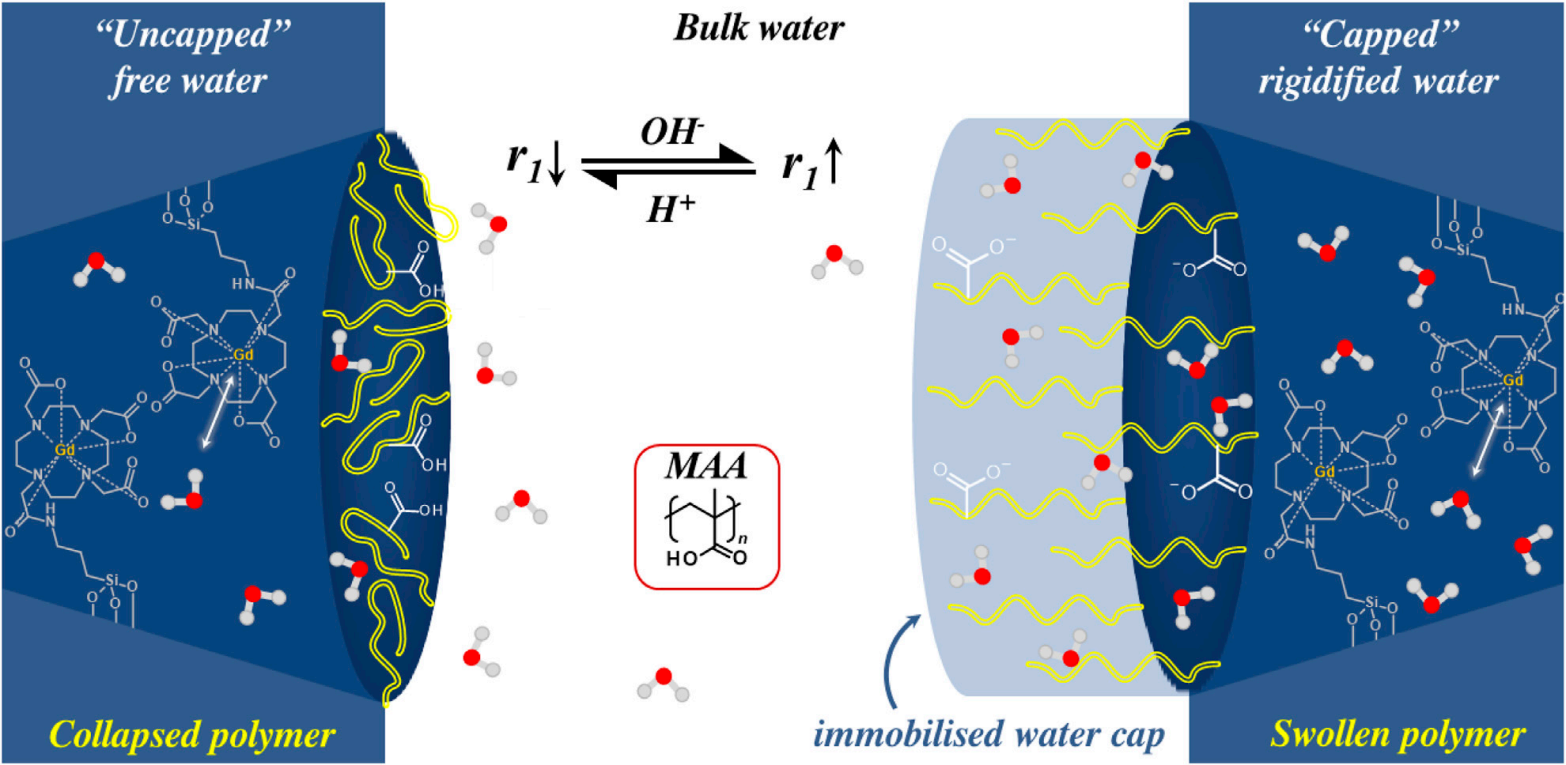
A schematic illustrating a pH-responsive *T*_1_ switch as observed with pMAA-Gd-MSNs. The pMAA shell in its uncharged/collapsed conformation allows limited hydration that results in a moderate *r*_1_. When the pH traverses the p*K*_a_ of MAA, the polymer shell charges and swells, dramatically increasing local hydration. The so generated “water cap” (light blue shaded region) boosts *r*_1_ by > 180%.

In a similar work by Yuan *et al.*, Gd-DOTA doped MSNs were surface modified with the pH-responsive poly (methacrylic acid) (pMAA), generated by use of a surface-initiated RAFT (SI-RAFT) polymerisation process (Figure 7). (Yuan et al., 2023) Notably, introduction of the polymer coat enabled *τ*_M_ to be modulated, directly influencing relaxivity. In this case, the reversibly charging polymer is not acting as a physical barrier to water access but as a potent hydrogen bond acceptor in the charged state; this is proposed to then modulate the relative viscosity of the local water pool and an SBM-theory supported promotion of relaxivity (Yuan et al., 2023; Ju et al., 2007; Sachar et al., 2020). The *r*_1_ relaxivity was assessed at 1.4 T, across a pH range of 4.0–9.0, with a significant switch of >180% ( $\Delta r_{1}$ = 30.3 ± 3.2 mM^−1^ s^−1^) as the ${pK}_{a}$ of pMAA was traversed. As these values exceed the theoretical maximum for the inner sphere contributions to total relaxivity, the shifts were assigned to increased outer sphere effects (De León-Rodríguez et al., 2015).

It is clear from these that suitable polymers, either electrostatically or covalently associated with NPs within inorganic-organic hybrid architectures, are able to support significant (>130%) pH-dependent enhancements in relaxivity upon traversal of their ${pK}_{a}$ , by virtue of conformational and/or electrostatic changes at individual monomer units. Only those approaches which employ covalent surface-bound polymers can support a reversible switch in image contrast upon cycling of bulk pH. The customisable nature of such polymer coatings, empowered through monomer choice, showcases the generation of inorganic-polymer hybrids as an attractive method by which a range of (multi)stimuli-responsive (*e.g.,* pH in combination with redox/light/enzyme) CAs could be considered. The so-generated responsive imaging probes, tailored to be responsive to a biological condition of interest, would enrich our understanding of disease pathology.

## 3 Paramagnetic organic nanoparticles

### 3.1 Polymer micelles

**FIGURE 8:**
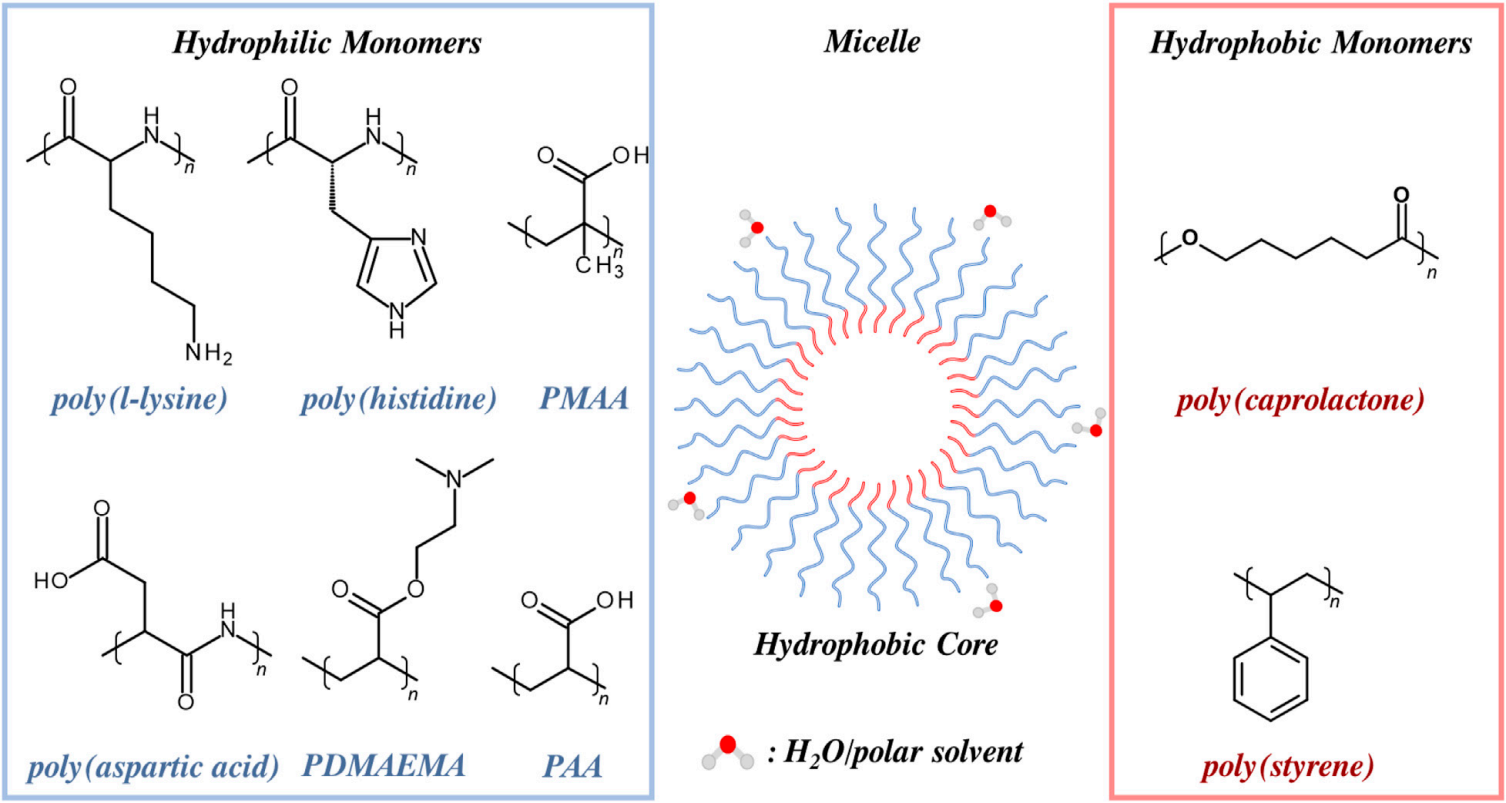
Examples of hydrophobic and hydrophilic polymers commonly utilised in micelle generation (hydrophobic core shown). Micelle surface functionality can be tuned through monomer selection. Carboxylic acid containing monomers (PAA, PMAA, PDMAEMA) and amino acid monomers (poly (aspartic acid), poly (l-lysine), poly (Histidine)) can be utilised to generate a physiologically-relevant pH-response. Hydrophobic monomers (PCL and PS) are also displayed.

Polymer micelles are typically composed of block co-polymers, containing both a hydrophobic and hydrophilic repeating unit, which self-assemble into micelles on suitable solvent exposure (Figure 8). (Yorulmaz Avsar et al., 2019) Hydrophilic blocks will naturally form the solubilising and stabilising micellular shell in aqueous conditions, with the hydrophobic block associating with the micelle core. Amphiphilic block-copolymers have been widely investigated as platforms for contrast agents, drug delivery, and catalysis due to their high associated biocompatibility, improved colloidal stabilities over non-modified inorganic nanoparticle analogues (*e.g.*, MSNs and IONPs), customisability, and tissue penetration ability (Deng et al., 2021). Polymer micelles are, for example, known to be able to capitalise on the tumour site accumulation characteristics of the EPR effect whilst avoiding glomerular filtration, promoting beneficially elongated blood retention times *in vivo* (Svenson, 2012). Physical properties, including the size and shape of the polymer micelle can be easily customised at the point of synthesis. They can be doped with a paramagnetic contrast agent such as Gd-DOTA through strand covalent attachment, or through physical entrapment within the micellar core (Kazunori et al., 1993). As prior discussed in Section 1.3., polymer micelles also offer improved relaxivities over their molecular analogues since the anchored or entrapped MR-active complexes have notably elongated rotational correlation times compared to the normal rotational characteristics of a molecular chelate (Lux and Sherry, 2018; Li et al., 2012).

**FIGURE 9:**
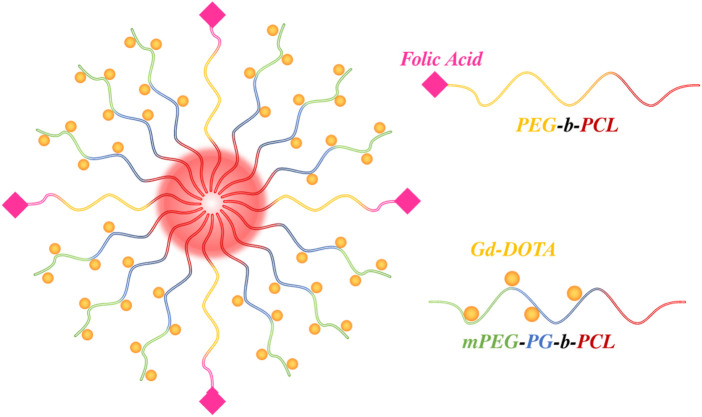
Schematic illustrating the micelle architecture employed by Cao *et al.* where MRI active Gd-DOTA moieties were tethered to individual mPEG-PG-*b*-PCL strands. Conjugation of folic acid to PEG-*b*-PCL polymeric strands enabled an active targeting capability (Yuan et al., 2025).

In one such example, Cao *et al.* synthesised amphiphilic block co-polymers containing methoxy poly (ethylene glycol)-polyglycerol (Gd-DOTA)-*b*-polycaprolactone (mPEG-PG (Gd-DOTA)-*b*-PCL) and folate terminated PEG-*b*-PCL which generated a micellular nanoparticle when mixed in a 4:1 ratio (Figure 9). (Cao et al., 2017) Conjugation of Gd-DOTA to the mPEG-PG-b-PCL polymer strands supported a significant (rotationally-enabled) enhancement in MRI contrast, (corresponding $r_{1}$ = 14.01 mM^-1^ s^-1^ at 0.5 T, a >250% improvement over traditional clinically employed Magnevist). Folate was chosen for its tumour targeting capability, facilitating rapid internalisation of the micelles by receptor-mediated endocytosis, common for tissues that overexpress folate receptors, such as cancer (Kularatne and Low, 2010; Wang et al., 2023).

**FIGURE 10:**
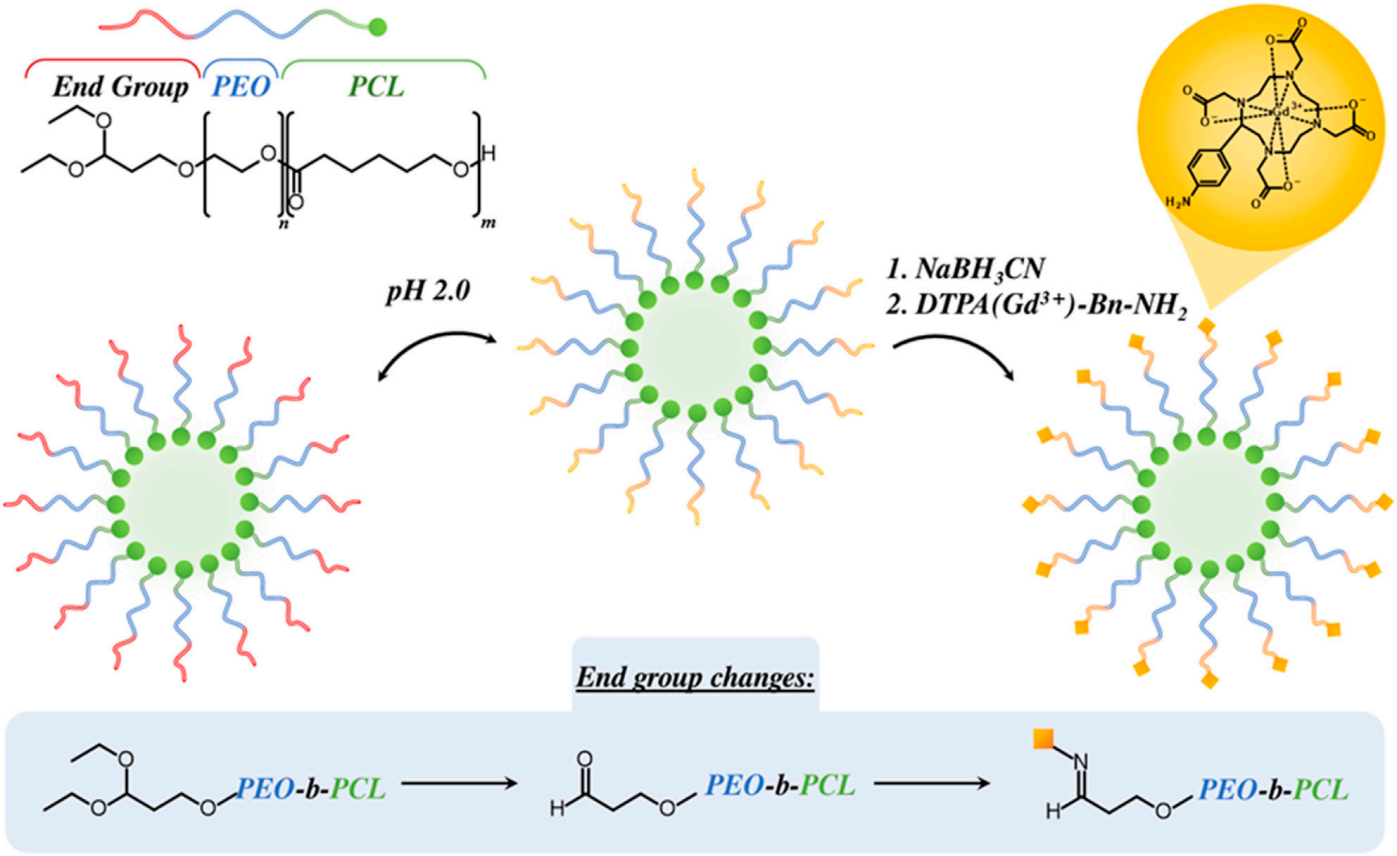
Schematised design of an amphiphilic block co-polymer poly (ethylene oxide)-b-poly (caprolactone) (PEO-b-PCL) and its self-assembly in solution. The polymer end group is subsequently modified to allow the tethering of a (Gd-DTPA) derivative containing a benzylamine moiety (DTPA (Gd^3+^)-Bn-NH_2_) by reductive amination using sodium cyanoborohydride (NaBH_3_CN) (Grogna et al., 2010).

While PEG is often chosen as the hydrophilic micellular component, poly (ethylene oxide) has also been reported. For example, Grogna *et al.* designed polymer micelles composed of poly (ethylene oxide)-*b-*poly (caprolactone) (PEO-*b*-PCL) as illustrated in Figure 10 (Grogna et al., 2010). A Gd-diethylenetriaminepentaacetic acid (Gd-DTPA) derivative containing a benzylamine moiety was subsequently bound to the PEO block by a reductive amination process at the integrated amine groups. In this work, the effect of polymer strand flexibility on relaxivity was examined by investigating differences in $r_{1}$ through polymer strand length (1,300 < M_n_ < 4,000). It was observed that shorter polymer strands yielded the highest $r_{1}$ (11.9 mM^-1^s^-1^), ascribed to a more restricted free rotation of the Gd-DTPA probe and associated elongated $\tau_{R}$ compared to the longer polymer strand micelles.

In terms of particle composition, monomers can be chosen to generate a micelle that is responsive to a specific environmental stimulus, such as deviations in pH or temperature. In the former, the selected monomer contains ionisable functional groups (*e.g.,* carboxylic acid or amine) which have an associated ${pK}_{a}$ that falls within a physiologically relevant range (pH 6.0–8.0). Upon traversal of the associated pH the native nanoparticulate structure can either conformational switch, or fragment entirely, as ionisable polymer blocks charge. Such changes can be used to modify parameters that control the (contrast-generating) relaxivity such as accessibility of water to the paramagnetic centres, and the rotational freedom order parameter (*F* ^2^), the latter describing the degree of independence of the paramagnetic complex rotation from the particulate scaffold. Examples of monomers utilised within assemblies include acrylic acid (AA), methacrylic acid (MAA), or dimethyl aminoethyl methacrylate (DMAEMA), all of which exhibit altered physicochemical properties at accessible, and relevant solution pH. Further examples can be generated from polymers comprised of amino acids, such as poly (aspartic acid), poly (L-lysine) and poly (histidine), synthesised from their respective N-carboxyanhydrides. These exhibit a pH-response through protonation/deprotonation of free carboxylic acid/amine groups, as well as through acid-regulated bond cleavage, and are additionally attractive as a result of their high associated biocompatibility (Yuan et al., 2025; Guo et al., 2020). Nitroimidazole derivatives and disulfide bridges can be post-synthetically added to the polymer strands, enabling demicellisation in reduction-sensitive environments, *e.g.*, tumour cells (Xu et al., 2022).

**FIGURE 11:**
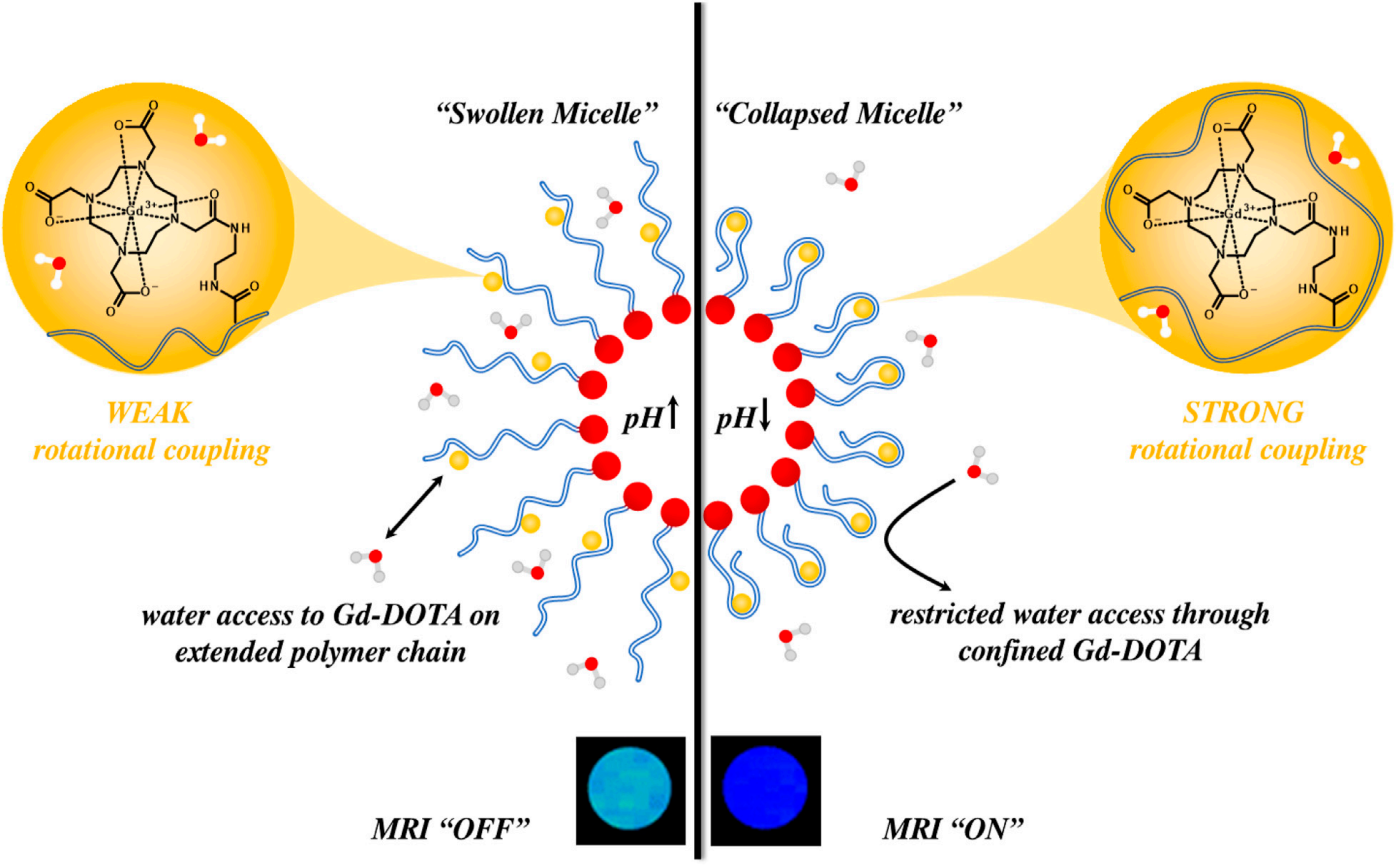
A schematic example illustrating how pH might influence the associated MRI signal for pH-responsive Gd-DO3A tethered micelles. At low pH, Gd-DO3A moieties are confined within polymer strands, displaying strong rotational coupling to the core of the micelle, shortening $T_{1}$ , and improving contrast. As pH increases, the PAA strands become deprotonated, electrostatically repel, and extend in solution. The rotational coupling of the chelate to the micelle core is lost, $T_{1}$ is longer, which results in a switch “off” MRI signal intensity.

A more detailed theoretical consideration of the effects of modulating chelate rotational rigidity in these structures was explored further in work by Ellis *et al.* Here, poly (acrylic acid)-*b*-poly (styrene) (PAA-*b*-PS) block copolymers were synthesised, with individual PAA polymer strands subsequently functionalised with Gd-DO3A by amide coupling (Ellis et al., 2023). The PAA segment of the micelle remains largely deprotonated at normal physiological pH (7.1–7.4), above the ${pK}_{a}$ of PAA, occupying an “extended” conformation since adjacent carboxylate groups electrostatically repel. As environmental pH falls below the ${pK}_{a}$ of PAA, the carboxylate groups are protonated, PAA strands become much more mechanically coupled to the micelle core, with relaxivity significantly increasing as the rotational characteristics of Gd-DO3A became akin to the nanoparticle scaffold. This caused a notable switch in $r_{1}$ , specifically $∆r_{1}$ = 9.70 mM^-1^ s^-1^ at 1.4 T as pH decreased from pH 7.5 to pH 4.0, an observation that was fully reversible. The order parameter was considered central to this. When F^2^ is equal to 0, the rotation of the paramagnetic complex is entirely independent of particle rotation, and when F^2^ is equal to one the complex is unable to rotate independently of the particle, *i.e.*, the mechanical coupling is “absolute” (Carniato et al., 2010). A F^2^ closer to one would be observed for rigid inorganic nanoparticles, such as small-pore MSNs or tightly contracted polymer micelles, and is associated with an increase in the local rotational correlation time of the paramagnetic centre, and therefore, by extension, $r_{1}$ . By designing a nanoparticle configuration that supports a locally triggered change in paramagnet-chelate rotation through polymer strand rigidity (see Figure 11), dramatic switches in $r_{1}$ can, then, be achieved.

**FIGURE 12:**
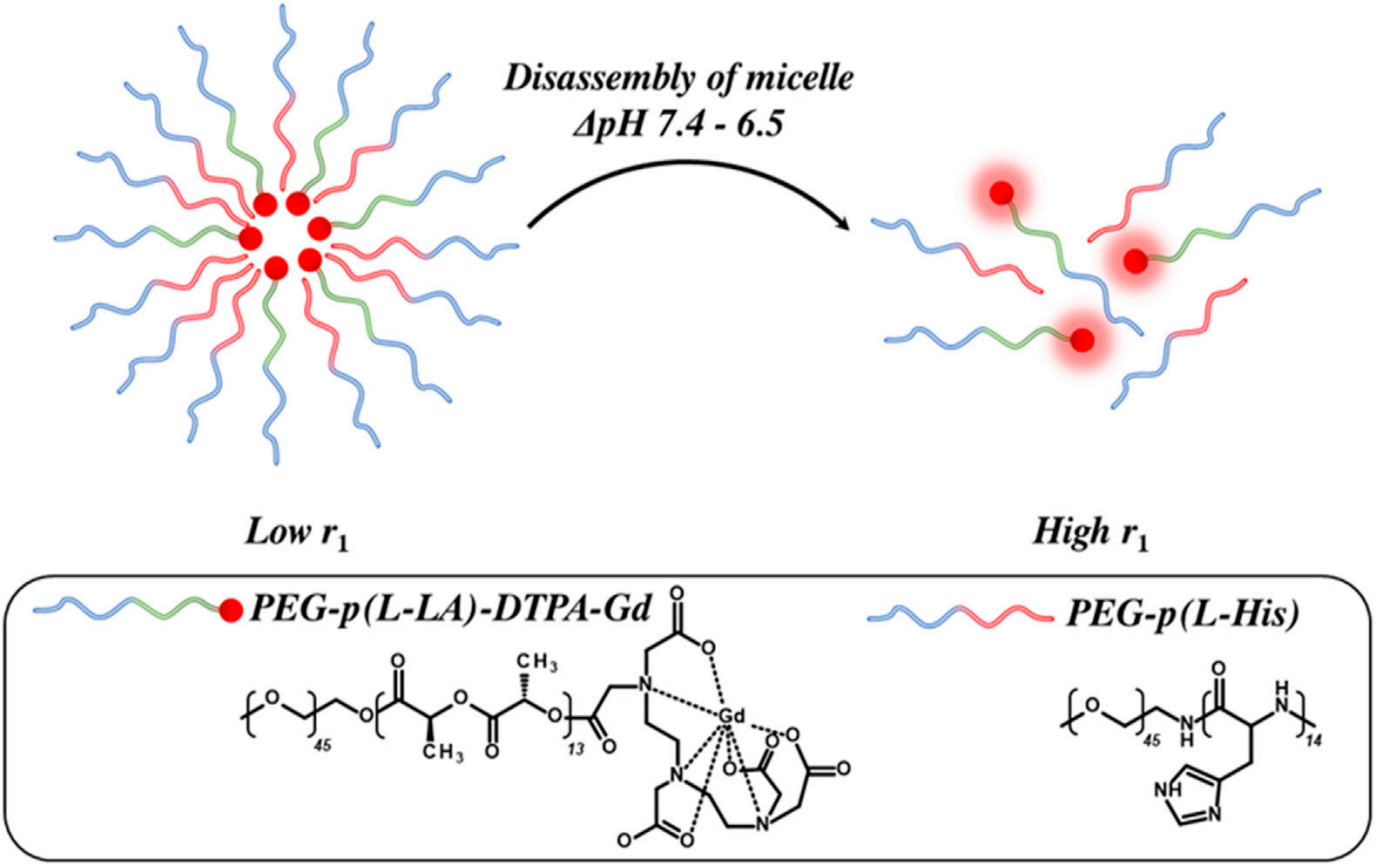
A schematic illustrating amphiphilic block copolymer methoxy poly (ethylene glycol)-*b*-poly (L-His) (PEG-p (L-His)), and methoxy poly (ethylene glycol)-*b*-poly (L-lactic acid)-diethylenetriaminopentaacetic acid dianhydride-gadolinium (PEG-p (L-LA)-DTPA-Gd) micelles. At physiological pH (7.4) the block copolymers self-assemble to generate colloidally stable micelles that fragment below the ${pK}_{a}$ of p (L-His), improving water accessibility to the core confined Gd-chelate.

While the above examples demonstrate a generalised reversible swelling approach to modulate generated image contrast, as noted, a destructive (irreversible) fragmentation approach can also be employed to modulate relaxivity. In work by Kim *et al.*, for example, block copolymers comprised of PEG-*b*-poly (L-lactic acid), tethered to Gd-pentetic acid (DTPA) and PEG-*b*-poly (L-Histidine) (PEG-*b*-p (L-His)), for pH-sensitivity, were designed that self-assembled to form micelles in aqueous solution (Figure 12). (Kim et al., 2014) At physiological pH (=7.4) the micelles retained a uniform and stable morphology, fragmenting on exposure to an acidic environment below the ${pK}_{a}$ of p (L-His) ( ${pK}_{a}$ = 6.8) as the imidazole groups protonate and undergo a hydrophobic to hydrophilic transition. The prior confined Gd-DTPA moieties, with initially highly-restricted water access, are released on fragmentation, improving $\tau_{M}$ , and resulting in a productive switch in $r_{1}$ ( $∆r_{1}$ = 3.45 mM^-1^ s^-1^ at 4.7 T).

Physical and downstream relaxometric changes in purely organic nanoparticles may also be triggered by exposure to a reducing environment, specifically GSH levels present in cancer are typically 2–5 times higher than healthy tissue, with an intracellular concentration ranging from 1–10 mM (Ge et al., 2024; Ding et al., 2021). Polymeric nanoparticles with engineered disulphide motifs can, specifically, support a responsive character through bond reductive cleavage on exposure to, *e.g.*, elevated GSH, triggering disintegration of prior cross-linked polymeric strands and exposing encapsulated MR active agents such as Gd-chelates. This engineering has, for example, been demonstrated in work by Sigg *et al.*, with the reductive cleavage of amphiphilic heparin-poly (dimethylsiloxane) (hepPDMS) block-copolymers, complexed with Gd^3+^ (Sigg et al., 2016). In this work, polymer hepPDMS strands were co-assembled with an integrated disulfide-linked peptide (peptide sequence *H*_*2*_ *N-*H_3_-X-[W-dL]_3_-W*-CONH*_*2*_) to form micelles, the latter containing the reducible motif X = (-CH_2_)_2_-S-S-(CH_2_)_2_-NH-CO-(CH_2_)_2_-CO-) that connects oligohistidine (H3SSgT) and hydrophobic L-tryptophan-D-leucine units. This reducible linker restrained the oligohistidine from extending into solution and exposing the integrated paramagnetic chelates, limiting water accessibility. Exposure to the reducing agent dithiothreitol (DTT) was observed to cleave the cross-links, resulting in improved water flux to the Gd^3+^ motifs, and enhancing associated image contrast ( $\Delta r_{1}$ = 10.20 mM^-1^ s^-1^ at 3 T).

### 3.2 Stimuli-responsive liposome CAs

**FIGURE 13:**
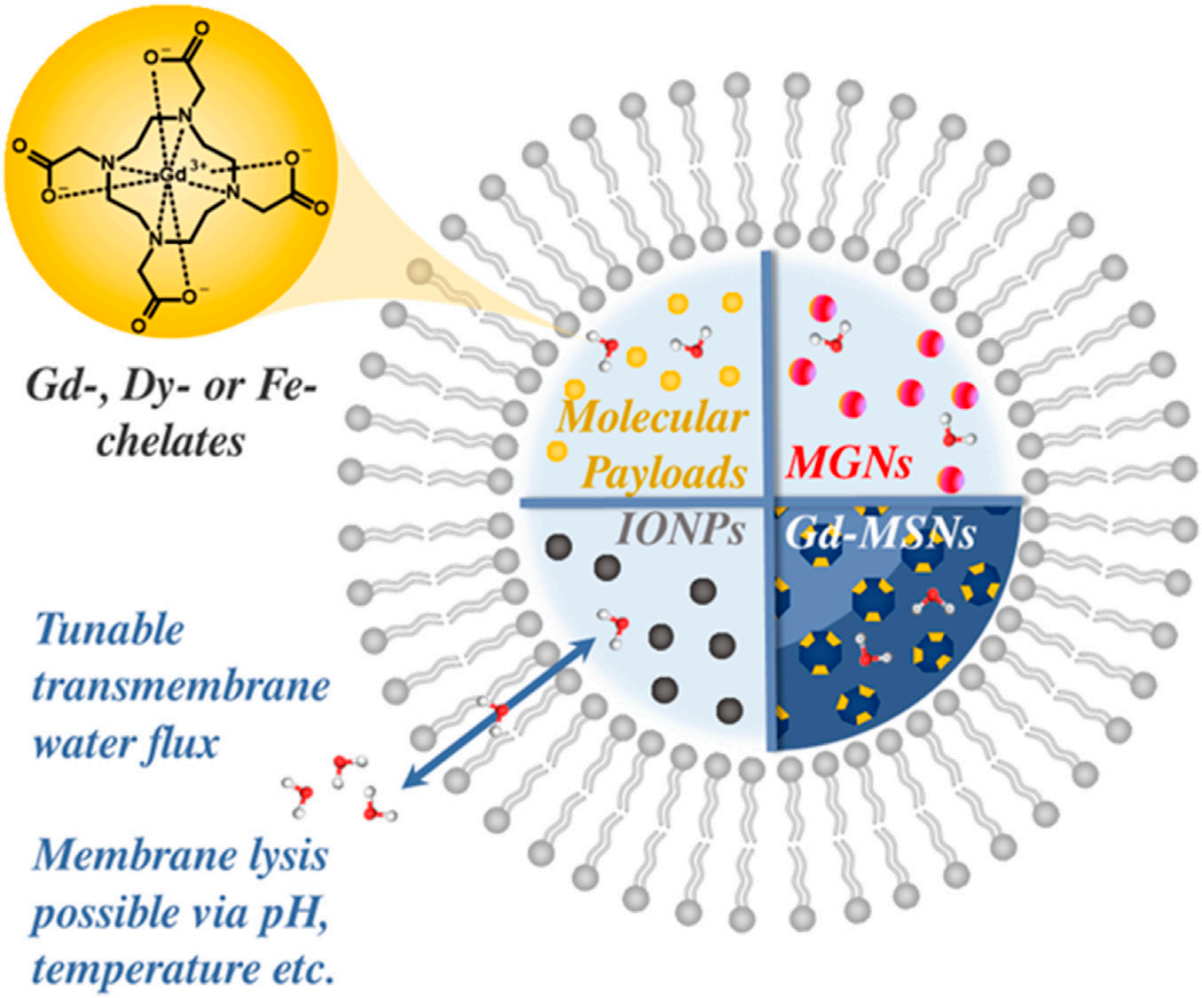
Phospholipid bilayers allow for the encapsulation of $T_{1}$ or $T_{2}$ active payloads, such as molecular chelates (*e.g.*, Gd-DOTA) or MR-active nanoparticles (Gd-MSNs, IONPs or MGNs). The rate of transmembrane water flux can be modulated in generating a responsive liposomal-based CA. Removal of the phospholipid membrane through lysis, or integration of transporter motifs that can retrospectively restore water flux, can enable a means by which relaxivity is modulated.

Liposomes are inherently biomimetic as a result of their vesicular structure, comprising a synthetic amphiphilic bilayer membrane. They offer high levels of colloidal stability, advantageous circulation half-lives, and beneficial *in vivo* degradation (Mulder et al., 2006; Rideau et al., 2018). These simple models of a cell can be productively interfaced with contrast generation by the encapsulation of MRI-active payloads (typically Gd- (Ayyagari et al., 2006), Dy- (Castelli et al., 2009) or Fe- (Chowdhury et al., 2023) chelates). Nanoparticulate probes, such as IONPs (Cardellini et al., 2024; Li Q. et al., 2022; Zhang N. et al., 2022; Waqar et al., 2022), quantum dots (QDs) (Xu et al., 2018), Mn_3_O_4_ nanoparticles (MGNs) (Thomas et al., 2023) or Gd-MSNs (Sun et al., 2018), can also be trapped with the hydrophilic core (Figure 13). (Soenen et al., 2011) Accommodation of these MRI active probes within liposomal scaffolds prevents non-specific interactions with serum proteins, offering protection against degradation during *in vivo* delivery, whilst additionally reducing opsonisation of the probe by RES (reticuloendothelial system) processes (Mulder et al., 2006; Zhang J. et al., 2022). In terms of clinical translatability, liposomes represent an established formulation for cancer nanomedicines, with Doxil (doxorubicin encapsulated within a PEGylated LUV) approved for oncology treatment since the 1990s (Cao et al., 2017; Wang et al., 2018).

**FIGURE 14:**
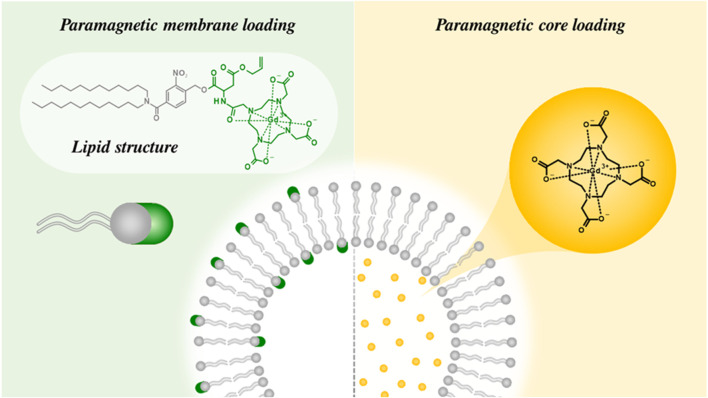
A schematic illustrating the means by which liposomes can be modified to be MR-active. Gd-chelates can either be encapsulated as a payload within the aqueous internal pool or covalently tethered to the phospholipid structure of the liposomal membrane (Liu C. et al., 2019).

Liposomes, by their nature, offer a platform through which responsive MR contrast, in the presence of stimuli such as pH (Løkling et al., 2001; Torres et al., 2011), temperature (Zhang et al., 2015; Kono et al., 2011), ultrasound (Kim et al., 2016) and/or light (Reeßing et al., 2019; Liu et al., 2022) can be achieved, as internally doped liposomes are initially in an 'MR silent’ state due to the limited water diffusion through the hydrophobic membrane. Inherent in derived CAs is an ability for this natural water barrier to be modulated. The magnitude of responsivity (relaxivity switch) may be increased by further improving the contrast ‘off-state’ to be more MRI ‘silent’ in nature, achieved by reducing the rate of water diffusion or bilayer permeability, e.g., through greater cholesterol loading (Saito and Shinoda, 2011). Central to this also is noting that inorganic imaging agents can either be readily encapsulated within the hydrophilic core of the vesicle or tethered to the bilayer itself (see Figure 14). In the latter, Gd-chelates are appended to the phospholipid hydrophilic heads and MR contrast generating. This provides a platform whereby relaxivity can be modulated by cleaving the pendant MR-responsive arm and modulating *τ*_R_ (Accardo et al., 2009). This is, once again, a reflection of the dramatic change in tumbling rate of a ∼100 nm liposomal structure and a free Gd-chelate (∼1 nm hydrodynamic size).

**FIGURE 15:**
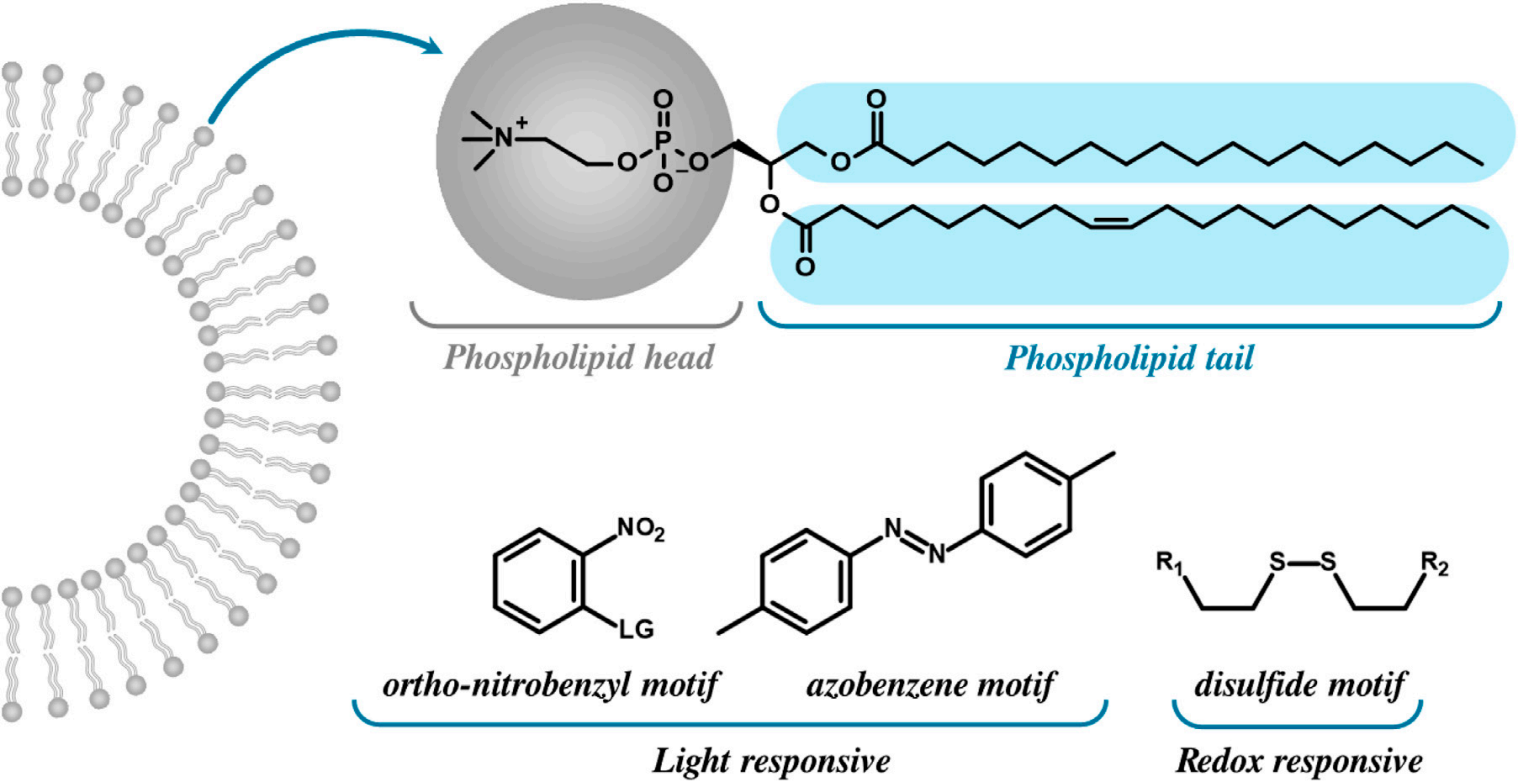
Bilayer structures can be chemically modified to become stimuli responsive in nature by incorporating light or redox responsive motifs into the phospholipid heads or tails. This is generally achieved by using a linker which can be cleaved upon exposure to certain stimuli, for example, the reduction of disulfides by TCEP (tris(2-carboxyethyl)phosphine hydrochloride) (Yao et al., 2021). Whilst integrated ortho-nitrobenzyl and azobenzene motifs can be reduced under certain conditions, as they are primarily light-responsive in nature. Azobenzenes integrated into phospholipid tails, for example, undergo E/Z photoswitching (Simon et al., 2022), altering the membrane permeability/diffusive water access. Phospholipid heads modified with ortho-nitrobenzyl groups, as an alternative, undergo photolysis and resultant cleavage of the leaving group such as a tethered paramagnet (Yao et al., 2021).

Liposomal MR responsivity may therefore be realised in two ways: destructively through lysis or fragmentation of the bilayer, triggering the controlled release of the *T*_1_ and/or *T*_2_ active payload, or non-destructively through selectively switching the permeability or transmembrane water flux (Mura et al., 2013; Nedyalkova et al., 2017; Fossheim et al., 1999). The latter is different to the mechanisms prior discussed for responsive micelle contrast generation in Section 3.1 where relaxivity is switched by either inducing a conformational change where local environment differs from the norm, or by inducing fragmentation entirely. The destructive approach in liposomes is generally used to achieve pH- or temperature-responsivity at regions of disease (generally acidic conditions and thermal stress), greatly switching water flux to initially integrated MR-active chelates (Yao et al., 2021). MR responsivity can also be achieved by chemically modifying hydrophobic phospholipid tails within the bilayer to introduce either stimuli-switchable, *i.e.,* altered membrane permeability, or stimuli-cleavable, *i.e.,* directly impacting *τ*_R_, motifs (Figure 15).

In an example of a triggered manipulation of liposome membrane permeability, Simon *et al.* generated switchable vesicles by introducing azo functionalities within phospholipid tails to enable UV-responsive MRI contrast (Pitchaimani et al., 2016). This tuneable membrane permeability specifically leveraged the structural impact of the azobenzene stereoisomerism with the cis (Z) isomeric form (produced through 370 nm irradiation) disrupting phospholipid packing, leading to an enhanced membrane permeability to water. This optical switching was demonstrated to be reversible, displaying UV-switchable MR contrast between MRI 'on' and ‘off’ states across three cycles.

Hyperthermia-responsive liposomes can be produced by using dipalmitoyl-phosphatidylcholine (DPPC), as demonstrated by Alawak et al. (2020). Here, MR active DPPC liposomes were shown to modulate diffusive water access to core-confined Gd-DTPA, with the thermo-responsive DPPC ultimately imparting a responsive $r_{1}$ capacity ( $\Delta r_{1}$ = 0.91 mM^-1^ s^-1^). As temperature was increased from 37.8°C, a DPPC gel-liquid phase transition (to a progressively more disordered state at T = 41.3 °C), resulted in the lipid membrane 'melting', altering the inherent bilayer permeability, switching on contrast with concurrent doxorubicin release from the micelle core. Alternatively, the liposomal membrane can be fragmented through external deviations in pH. This was demonstrated in work by Li *et al.* who encapsulated Cu-doped IONPs (size 15 ± 5 nm) within the core of hydrogenated soy phosphatidylcholine (HSPC) liposomes. Acid-triggered biodegradation (pH < 7.0) enabled switchable *T*_2_ contrast as the lipid head group became protonated at low pH, destabilising the bilayer and resulting in membrane lysis and a darkening of $T_{2}$ -weighted MR images (Thomas et al., 2023; Paliwal et al., 2015).

Irreversible rupture-based approaches to triggered MR contrast change can also be achieved through acid/UV induced bond cleavage from liposomal peripheries. In a switch “off” example of this, Liu *et al.* introduced a Gd-DTPA and o-nitrobenzyl (ONB) modified phospholipid within a tightly-packed vesicular membrane (Liu C. et al., 2019). In this work, the liposomes were also internally doped with DOX for concurrent drug delivery. Initially, the covalent tethering of the Gd-chelates to the phospholipids significantly reduced (as we have noted previously) the tumbling rate of the Gd^3+^ moiety compared with a typical Gd-chelate (modulating *τ*_R_, boosting initial *r*_1_). This example, then, presents dual-trigger release/hydrolysis capabilities (pH and light), whereby subsequent UV cleavage of the ONB results in the release of Gd-DTPA and a concurrent decrease in rotational correlation time, *τ*_R_ of the Gd moiety, and relaxometric switch.

The abnormal local concentrations of oxidising species (and reduced pH) at tumour tissue can be exploited in the generation of redox-responsive MRI contrast, which can report on, for example, the elevated reactive oxygen species (ROS) presence at such sites. In work by Thomas *et al.* a pH- and ROS-responsive probe was developed based on the encapsulation of Mn_3_O_4_ nanoparticles (size 20–30 nm) within PEGylated liposomes (composed of 1,2-distearoyl-sn-glycero-3-phosphoethanolamine-PEG, DPPC, and cholesterol) (Thomas et al., 2023). Switchable *T*_1_ contrast (enhancement) was generated here upon reduction of the Mn_3_O_4_ nanoparticles, the production of highly paramagnetic Mn^2+^ and a $\Delta r_{1}$ = 3.84 mM^-1^ s^-1^ at 3 T.

These approaches so far generally follow one of two mechanistic approaches to modulate relaxivity in the presence of a particular external stimulus, depending on whether the liposome is either membrane modified or internally doped with the MRI signal-generator moiety. In the case of the former, a specific change in *τ*_R_ results from covalent cleavage of the paramagnetic chelate. Alternatively, for the latter, the initially very limited diffusive water access across the lipid bilayer is reversed by altering membrane permeability, enhancing $\tau_{m}$ , in a manner that may or may not be reversible (e.g., a destructive or geometric isomerism switch of bilayer motifs).

**FIGURE 16:**
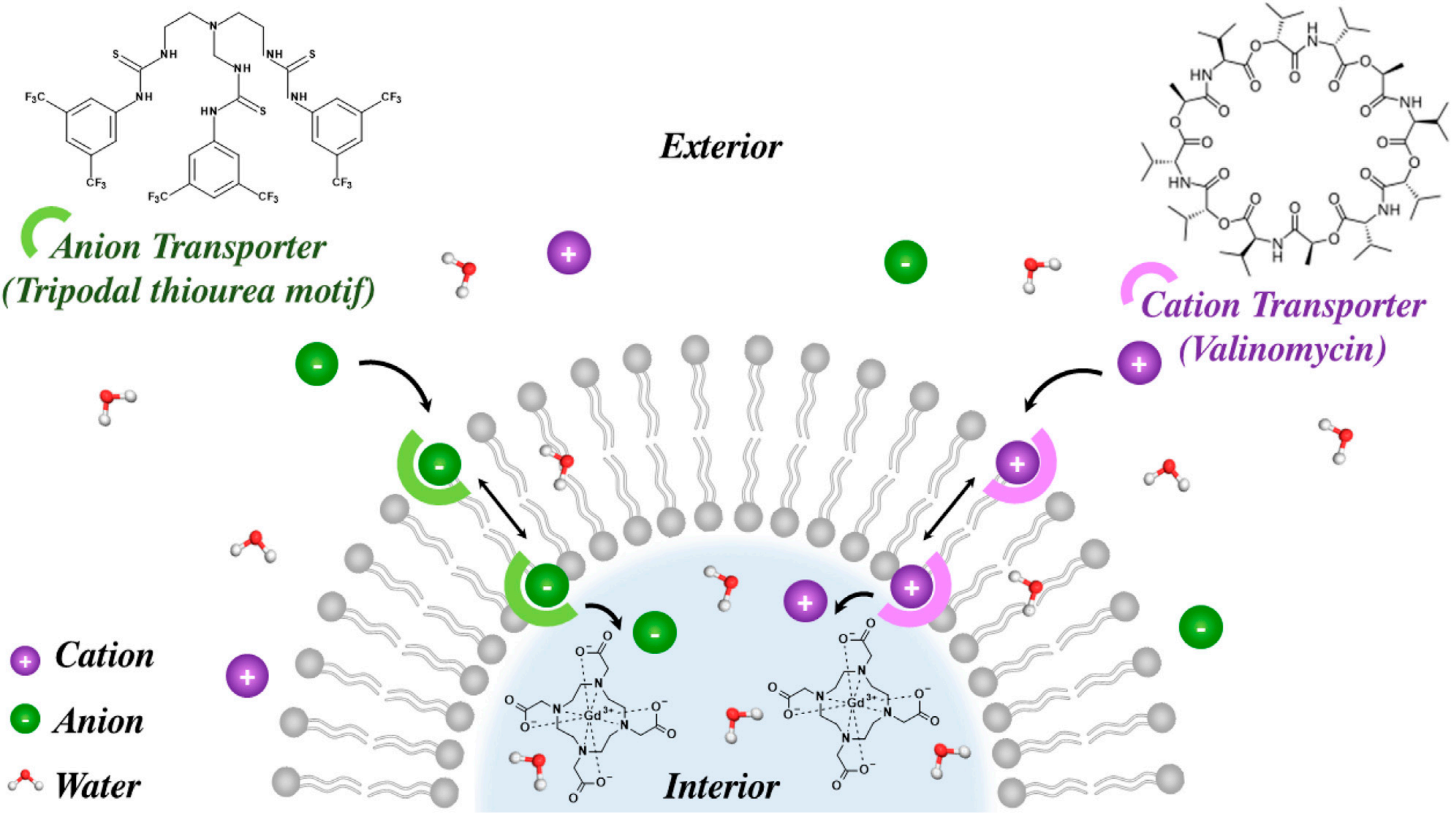
Simultaneous transfer of M^+^ and X^−^ upon co-integration of a tripodal thiourea motif (an anion transporter) and valinomycin results in an elevation of cross-membrane water flux and hence measured relaxivity by both $T_{1}$ measurements and MRI phantoms. This is dominated by the increase in water flux across the initially highly hydrophobic bilayer upon mobile carrier incorporation and resultant ion flux, giving a ∼60% switch in longitudinal relaxivity for Gd chelate doped liposomes.

In work by Duncan *et al.*, a more sophisticated triggered modulation in transmembrane water flux was used to generate *ion*-responsive contrast. Here cholesterol modified POPC liposomes internally doped with either Gd-DOTA or Gd-MSNs were generated (Duncan et al., 2024). This was followed by the dual integration of K^+^ and Cl^−^ selective ion carrier species, namely, valinomycin and a tripodal thiourea-based motifs, which could transport the desired ions across the bilayer; central here is noting that this ion transport is associated with significant associated water. Upon simultaneous transport of both cations and anions (ionic symport activation) the carrier-mediated passage of both M^+^ and X^−^ across the bilayer was enabled, as displayed in Figure 16. Concurrently, ion associated water flux is greatly enhanced, elevating the cross-membrane exchange rate, allowing notable modulations in MRI contrast to be observed in *T*_1_ weighted MRI maps (with *r*_1_ increasing by up to 200% dependent on the nature of the integrated paramagnet). Such variations in *r*_1_ were found to correlate with the selectivity of the cationophore and anionophore present and support, of course, an engineered ion responsive contrast generation.

Stimuli responsive liposomal MRI CAs can be further developed to incorporate dual-modal (*T*_1_-*T*_2_) MRI probes. An example of this was demonstrated by Zhou *et al.*, who investigated the NIR and H_2_O_2_ activated oxidation of ferrocenyl (Fc-) compounds, specifically the oxidation of Fc (hydrophobic) to Fc^+^ (hydrophilic) within ferrocenylseleno-modified Gd^3+^-doped liposomes through Fenton chemistry (Zhou et al., 2023). The associated transition from hydrophobic to hydrophilic (as Fc^+^) was proposed to improve local hydration and facilitate water exchange at both the Gd^3+^ and Fe^3+^ centres, switching both $r_{1}$ and $r_{2}$ with $\Delta r_{1}$ = 0.52 mM^-1^ s^-1^ and $\Delta r_{2}$ = 1.28 mM^-1^ s^-1^ recorded at 0.5 T.

Liposomal MRI CAs represent, then, an attractive organic scaffold where generated MR contrast can be specifically modulated by a programmable and wide variety of stimuli. Responsivity in these frameworks may be achieved both by non-reversible (*i.e.*, membrane lysis or peripheral cleavages of MRI active moieties) or reversible (water gating) means. Furthermore, such responsive probes have highly developed theranostic applications (*e.g.*, integration of the anti-cancer agent doxorubicin) in addition to a diagnostic approach (*r*_1_ and/or *r*_2_ active payloads) (Pitchaimani et al., 2016; Karimi et al., 2016). Given their biomimetic nature, and therefore high associated biocompatibilities, one can foresee these as emerging and potent theranostic agents.

## 4 Outlook and future work

Recent developments in nanoparticulate organic chemistry can be leveraged in the generation of contrast supporting agents that are highly responsive in specific physiologically-relevant environments.

Through all of these approaches, which employ organic/organically-coated nanoparticles, including those which are liposomal, micellar, dendritic and inorganic-polymer hybrid in nature, the importance in considering water access, exchange rate, and rotational correlation when designing a particular CA is emphasised. These platforms maintain the typical benefits of nanoparticulate CAs such as high tunability, morphological control, and the possibility of internal cargo loading. Additionally, as is generally true for nanoparticle-based MRI CAs, baseline relaxivity/generated contrast is greatly increased due to beneficially elongated rotational correlation characteristics that result in the tumbling of the paramagnetic-chelate becoming slowed and closer to the optimal Larmor frequency (as demonstrated by SBM theory). Inorganic-polymer hybrids include polymer amended IONPs and MSNs that can control associated image contrast through a considered manipulation of these same governing SBM parameters. Strategies include the irreversible aggregation/disaggregation of IONPs through control of the organic coating chemistry that can dramatically influence the magnetic susceptibility of the imaging probe, resulting in a significant switch in $r_{2}$ . Alternatively, the use of surface-polymer bound MSNs can be employed to controllably gate the release of an integrated MR-active cargo, restoring initially highly restricted (necessary) water access. This can be either through irreversible loss of electrostatic association to the particle surface as polymer ${pK}_{a}$ is traversed, or by reversible extension of individual polymers into solution through repulsion of adjacent charged strands (beneficially elongating $\tau_{D}$ / $\tau_{m}$ ).

Similar approaches are also used for the generation of responsive “purely organic” agents; the irreversible fragmentation of micellular and liposomal contrast agents under local stimulus is a common method to release core confined integrated MRI-active agents and restore water access, for example,. Here, the fragmentation is often caused by either a change in the ionisation state of the organic component, mediated through solution pH and ${pK}_{a}$ , or through reduction of cleavable bridges that include disulfide links. Alternatively, as is the case for polymer micelles, rotational characteristics of polymer-tethered paramagnetic chelates can be controllably, and often reversibly, altered through a modulation of polymer conformation. Specifically, extension of individual strands into solution, as ${pK}_{a}$ is traversed or disulfide bonds are cleaved, can result in a switch in relaxivity as mechanical coupling of the chelate to the nanoparticle core is lost and $\tau_{R}$ becomes dictated by rotation of the chelate. Liposomal agents have a long and potent history as therapeutic delivery systems but can also be powerfully integrated into environmentally-responsive contrast agents. With these water flux from bulk can be influenced by virtue of the (programmable) lipid bilayer which can provide a barrier to water exchange on rigidification (*e.g.,* by cholesterol doping), restorable by melting, distortion or through the integration of appropriate transporters (*e.g.*, solvated ion transporters).

To summarise, the marriage of inorganic magnetochemistry with organic functionality supports a colourful range of responsive CAs. These already integrate a diverse array of mechanistic approaches to switch *T*_1_ or *T*_2_ image contrast on exposure to a range of external environmental stimuli. Continued developments in this field look to generate highly biocompatible configurations supporting a significant switch ‘on’ in relaxivity under conditions which align with physiologically relevant microenvironments and associated diagnostic need. Going forwards, one can foresee that this combination of broad chemical know-how represents a potent means of enabling the high contrast imaging of specific pathology, potentially with integrated therapy.
